# Phospholipid Interconversion and Transport Are Altered in Glaucoma

**DOI:** 10.1096/fj.202504068R

**Published:** 2026-01-20

**Authors:** Genea Edwards, Ruminder Kaur, Anna Mueller, Noël Ziebarth, Richard K. Lee, Sanjoy K. Bhattacharya

**Affiliations:** ^1^ Department of Ophthalmology, Bascom Palmer Eye Institute University of Miami Miami Florida USA; ^2^ Department of Biochemistry and Molecular Biology University of Miami Miami Florida USA; ^3^ Miami Integrative Metabolomics Research Center University of Miami Miami Florida USA; ^4^ Department of Biomedical Engineering University of Miami Miami Florida USA

**Keywords:** aqueous humor, glaucoma, phospholipid, primary open‐angle glaucoma, trabecular meshwork

## Abstract

Glaucoma, the leading cause of irreversible blindness worldwide, is characterized by elevated intraocular pressure (IOP) resulting from impaired aqueous humor (AH) outflow through the trabecular meshwork (TM). We demonstrate altered phospholipids (PLs) levels and increased activity of the interconversion enzyme phosphatidylserine decarboxylase (PSD) in the TM of individuals with primary open‐angle glaucoma. Elevating PSD alone is sufficient to raise IOP in normotensive mice, whereas depleting PSD restores IOP in glaucomatous mice. Although certain PL classes are generally altered in glaucoma, specific PLs with minor structural variations within those classes exhibit variability in their levels in TM and AH, indicating a potential lipid transport abnormality. We identified a distinct set of ocular lipid species altered in glaucoma, implicating alterations in the levels and functionality of the lipid transporter ATP8B2. This flippase alone, however, does not seem to affect IOP but appears to amplify the effects of PSD.

## Graphical abstract

Decreased phosphatidylserine (PS) and increased phosphatidylethanolamine (PE) occur in both trabecular meshwork (TM) cells and aqueous humor due to aberrant phosphatidylserine decarboxylase (PSD) over‐expression, and variation in levels of individual PS or PE species differing by only a few acyl chains occurs due to impaired lipid transport due to decreased flippase ATP8B2 activity in primary open‐angle glaucoma. PSD modulation alone, but not ATP8B2, is sufficient to trigger intraocular pressure elevation, though the latter has an additive effect.

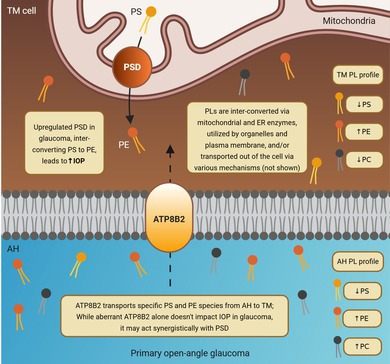

## 1 Introduction

Glaucoma is a group of irreversible blinding diseases affecting up to 80 million people worldwide [1]. The most prevalent type of this condition is primary open‐angle glaucoma (POAG). In general, glaucoma involves the loss of retinal ganglion cells and is often characterized by elevated intraocular pressure (IOP) due to increased resistance to the outflow of aqueous humor (AH) through the trabecular meshwork (TM). IOP homeostasis is an intricate mechanism through which ocular structures maintain a narrow range of pressure [2]. Large diurnal IOP fluctuations precede glaucomatous changes, indicating an IOP homeostasis dissolution early in POAG [3].

Phospholipids (PLs) are major structural and functional components of cell membranes, participating in a wide range of regulatory processes [4]. In mammalian cells, phosphatidylcholine (PC) is the most abundant phospholipid, followed by phosphatidylethanolamine (PE), phosphatidylinositol (PI), and phosphatidylserine (PS) [4, 5]. PLs are synthesized intracellularly through several connected pathways. Many of these pathways employ interconversion of different phospholipid classes and/or species [6]. Each class of PLs possesses distinct properties, giving rise to diverse outcomes [7, 8, 9, 10, 11]. Changing the ratio of PCs to other phospholipids, for instance, modifies cell membrane shape, permeability, and geometry [12, 13, 14, 15]. PE, on the other hand, is hypothesized to facilitate vesicular budding and membrane fusion [16]. PC and PE can be metabolized to release a plethora of bioactive molecules, such as arachidonic acid and diacylglycerol [17]. The negatively charged PS, largely located at the inner leaflet of the plasma membrane, is thought to serve as the electrostatic mediator for numerous proteins [6].

Normal AH carries a diverse PL repertoire originating from anterior segment structures [18]. The PLs in the TM, as well as mechanosensitive channels, directly experience pressure changes and are likely to participate in IOP homeostasis regulation [19, 20, 21, 22]. Long‐standing ocular hypertension may disrupt anterior segment PLs morphologically and functionally. Alternatively, PL alterations may contribute to elevated IOP. Significant changes in the PL composition of the TM, AH, and blood serum have been linked to glaucoma [23, 24, 25]. In advanced glaucoma, there is a significant PL level decrease in erythrocytes, which may serve as a disease severity biomarker [26]. In this paper, we investigate the mechanisms by which PL levels are impaired in glaucoma.

## 2 Materials and Methods

### 2.1 Human Donor Tissue and AH Samples

The human donor eyes were procured from Midwest Eye Banks, Cincinnati Eye Bank, and Florida Lions Eye Bank in Miami. TM tissue was dissected from donor eyes. Corneal transplant donor tissue was also procured from Mundorf Eye Center in Charlotte, NC. The AH samples were collected from the clinics of Drs. Richard Lee, James Banta, and Anna K. Junk under IRB‐approved protocols. The donor's details have been provided in Table S1. Samples from patients with glaucoma were included in the study if death to enucleation was less than 24 h for all experiments (except for Western blot analysis, where this criterion was relaxed for up to 32 h, based on our prior experimental experience) and for at least some details of static perimetry data were available indicating progressive visual field loss. The availability of some general medical history was also considered for inclusion. The exclusion criteria for patients were the presence of any other eye disease, especially the record of any other retinal diseases. Refractive error‐related issues or cataract were not part of the exclusion criteria.

### 2.2 Mass Spectrometric Lipid Profiling and Protein Quantification

High‐resolution mass spectrometric analysis of lipids extracted using the Bligh & Dyer method [27] after dissection of TM tissue was performed on a Q‐exactive instrument following fractionation on an Accela HPLC instrument (Appendix S1). Briefly, typically 100 μg tissue was used for extraction following our established protocols [23]. Lipids containing phase were carefully transferred to a fresh tube and solvent dried in a SpeedVac. Dried samples were typically redissolved in 20 μL of a mass spec compatible solvent prior to HPLC coupled with high resolution tandem mass spectrometry [23]. Identification and quantification of lipids were performed using LipidSearch software. For protein quantification, a set of iTRAQ 8plex Multiplex reagents was used to tag the peptides on the samples. Trypsin‐digested peptides fractionated on Easy nLC were analyzed using Q‐exactive and Proteome Discoverer following previously published protocols [28, 29].

### 2.3 Enzymatic Assays and Steady‐State Intermediate Measurements

The enzymatic activities for phospholipid conversion pathways were determined from 10 μg of total protein extract of control and glaucomatous TM tissue (*n* = 10 each) with suitable modification of assay methods compatible for mass spectrometric analysis of substrate and products.

### 2.4 Western Blot, ELISA, and PCR Analysis

Western blot, dot blot, and ELISA analyses utilized established protocols [30, 31]. Proteins were separated [10 μg proteins per lane for human TM/other ocular tissues and 5 μg proteins per lane for mice TM/other ocular tissues respectively unless stated otherwise] on a 4%–20% SDS‐PAGE and were transferred onto a PVDF membrane. RNA was isolated from TM nuclear extract and AH using the miniRNA extraction kit (Stratagene Inc.) according to the manufacturer's protocol.

### 2.5 IOP Measurements for Aqueous Humor Dynamics (AHD)/Outflow Pathway

IOP was measured in animals using a rebound tonometer [32] (Tonolab, Colonial Medical Supply). Animals were anesthetized by intraperitoneal injection of a ketamine/xylazine mixture. A subset of confirmatory IOP measurements was also performed using cannulation methods developed by Dr. Simon John [33, 34]. We also performed outflow facility assessment in a limited subset of mice using a multi‐level constant pressure perfusion method [35].

### 2.6 Thin‐Layer Chromatography (TLC) of Phospholipids

Phospholipids were separated by thin‐layer chromatography (TLC) on silica gel 60 F254 plates (Merck) with minor modifications from published protocols. Lipids were extracted from samples using the Bligh and Dyer method, dried under nitrogen, and dissolved in chloroform: methanol (2:1, v/v). Aliquots (1–5 μL) containing 0.1–2 μg lipid were applied 1 cm from the lower edge of pre‐washed plates and developed in chloroform: methanol: acetic acid: water (50:30:8:4, v/v/v/v) to approximately 8 cm from the origin. After air‐drying, lipids were visualized by either primuline staining (0.05% in acetone: water, 80:20, v/v) under UV illumination. A subset of confirmatory analysis was also performed by charring following spraying with 10% CuSO₄ in 8% H₃PO₄ and heating at 180°C for 10 min. Phospholipid species were identified by comparison to authentic standards (phosphatidylcholine, phosphatidylethanolamine, phosphatidylserine, and phosphatidylinositol procured from Avanti Polar Lipids), and relative band intensities were quantified by densitometry using ImageJ software. As needed, data were normalized to an internal standard (dipalmitoyl‐phosphatidylcholine) and expressed as relative abundance or mol% of total phospholipid.

### 2.7 Flippase Activity Assay

ATP8B2 flippase activity was assessed using a cell‐based NBD‐lipid uptake and dithionite quenching assay as previously described with minor modifications. Briefly, HEK293T cells transiently expressing human ATP8B2 and its CDC50A subunit were incubated with 2 μM NBD–phosphatidylserine (Avanti Polar Lipids) in Hanks' balanced salt solution (HBSS) containing 10 mM HEPES (pH 7.4) for 10 min at 25°C to allow lipid incorporation into the plasma membrane. Cells were washed and treated with 5% fatty‐acid‐free BSA in HBSS for 5 min to remove surface‐accessible lipid, followed by fluorescence quenching with 20 mM freshly prepared sodium dithionite in Tris (pH 9.0) for 5 min on ice to eliminate outer‐leaflet fluorescence. Residual fluorescence, representing internalized (flipped) NBD‐lipid, was measured by flow cytometry (excitation = 470 nm; emission = 530 nm). Parallel controls included vector‐only, ATP8B2 catalytic mutant (D454N), and ATP‐depleted cells (treated with 10 mM sodium orthovanadate and 10 mM EDTA). Data were expressed as percent dithionite‐resistant fluorescence relative to total signal before quenching and normalized to wild‐type ATP8B2 activity. All experiments were performed in triplicate, and results are presented as mean ± SD. Primary TM cells were used for ATP8B2 assay in an identical manner.

Protein–lipid photocrosslinking was performed using a photoactivatable lipid probe (diazirine‐ or benzophenone‐modified phospholipid; Avanti Polar Lipids or synthesized probe) incorporated into membranes either by incubation with live cells or by inclusion (0.5–2 mol%) during liposome formation/reconstitution. Cells or proteoliposomes were equilibrated in ice‐cold assay buffer, placed in a clear, uncovered 35–60 mm dish on ice ~5 cm below the Stratalinker light source (Stratagene/Agilent Stratalinker 1800 equipped with 365 nm bulbs), and irradiated at 365 nm (two 60–120 s pulses, total 2–4 min; optimize time empirically to maximize crosslinking while minimizing photodamage). Irradiation on ice minimized thermal drift and non‐specific damage; negative controls included samples lacking the photo‐probe, non‐irradiated samples, and samples containing an inactive protein mutant or non‐hydrolyzable nucleotide where appropriate. After UV exposure, reactions were quenched by immediate addition of ice‐cold quench buffer (e.g., 50 mM Tris pH 7.5, 150 mM NaCl) and solubilized in SDS sample buffer (or lysed for downstream enrichment), then analyzed by SDS–PAGE followed by in‐gel fluorescence (for directly fluorescent probes) or proteomic identification by LC–MS/MS analysis of excised bands. NBD‐lipids (NBD‐DSPS and NBD‐DSPE) were crosslinked with cell extracts in an identical manner as described above and found to work for protein‐lipid crosslinking. NBD‐Lipid crosslinked proteins were separated on 4%–15% or IEF 4–9 pH IEF PHAST gels and subjected to LC–MS/MS identification. All steps involving NBD‐lipids or photoactivatable reagents were performed protected from ambient light, dithionite/chemical quenching and reducing agents were avoided prior to analysis, and appropriate UV shielding and personal protective equipment were used. Stratalinker bulb type remained unchanged throughout the course of experiments. Key experimental parameters (probe identity and % mol, protein: lipid ratio for reconstitutions, irradiation distance and total exposure, and negative/positive controls) were optimized based on fluorescence estimated using NBD‐DSPS and kept unchanged. Three independent observers performed experiments and hence these parameters were recorded for each experiment to ensure reproducibility.

All methods used in these studies are described in greater detail in the accompanying supplemental methods. Study designs incorporated randomization. All reagents, including any cell line used, were subjected to in‐house authentication. All presented results are derived from at least three independent biological replicate experiments.

### 2.8 Statistics

Data are presented as mean ± SD or mean ± SEM as indicated. Statistical significance and P values were calculated using Student's 2‐tailed *t*‐test or ANOVA, with *p* < 0.05 and *p* < 0.01 considered statistically significant, *p* < 0.001 and *p* < 0.0001 considered highly significant.

### 2.9 Study Approval

All mice were used in accordance with an approved IACUC protocol of the University of Miami. Human cadaveric tissue or AH were procured without identifiers. The cadaveric samples collected without identifiers are exempted from IRB approval under NIH category 4. AH samples or any surgical tissues were procured under University of Miami IRB approved protocols.

## 3 Results

### 3.1 Phospholipid Interconversion Is Altered in Glaucoma

**FIGURE 1:**
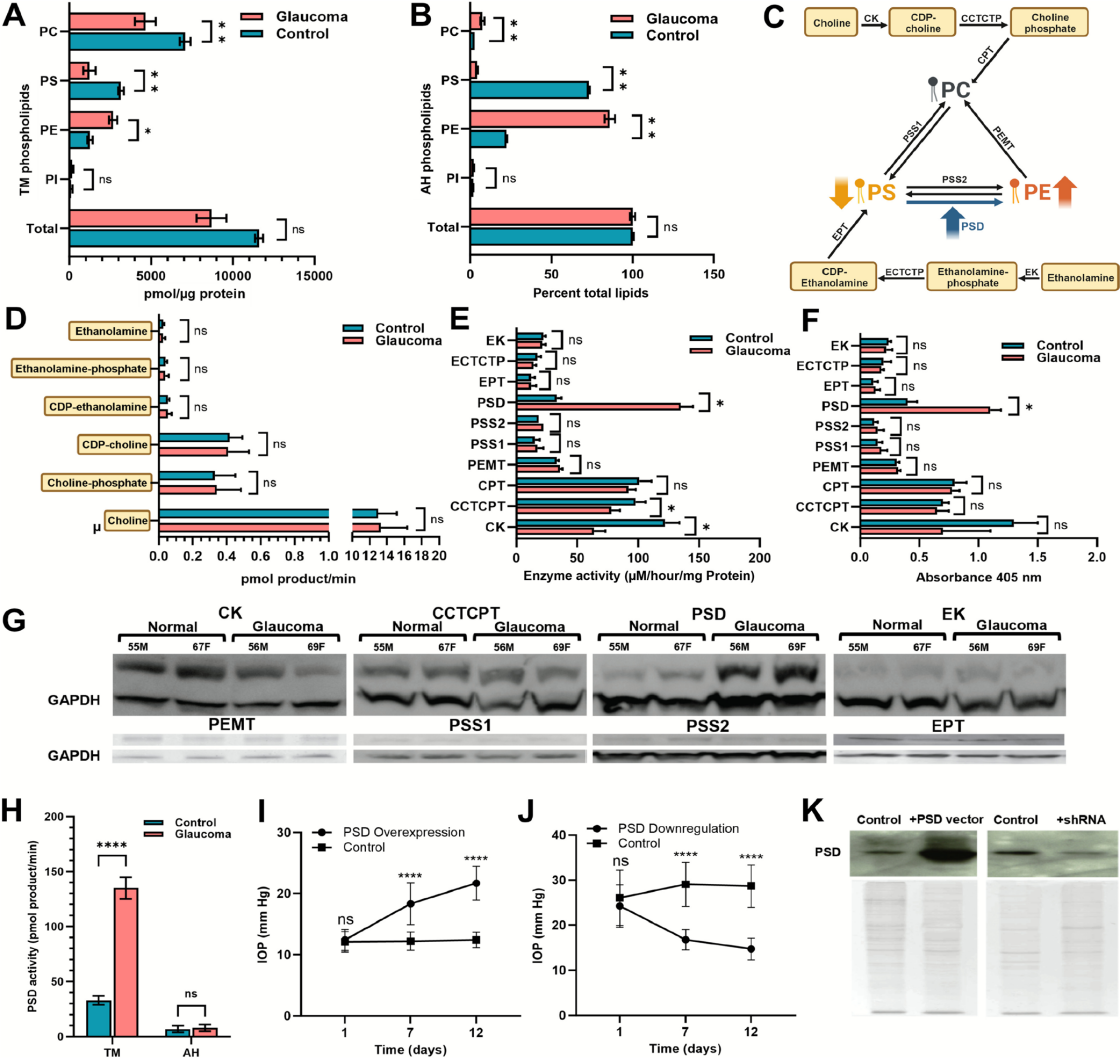
Phospholipid interconversion is aberrant in glaucoma. Phospholipid classes from (A) TM and (B) human AH were evaluated by mass spectrometry. Phosphatidylcholines: PC; Phosphatidylethanolamines: PE; Phosphatidylserines: PS; Phosphatidylinositols: PI, all phospholipids are depicted as total. AH lipid is expressed as a percent of total lipid (mean of three experiments) due to corresponding very low protein concentration. *n* = 40 each TM and AH was used for these experiments ANOVA was performed to determine significance. *p ≤ 0.05; **p ≤ 0.01, ns = not significant. (C) The biosynthetic pathway of phospholipids. Steady state level analyses of (D) phospholipid metabolite precursors and (E) conversion enzymes. (^14^C)choline, (^3^H)choline and ^32^P were used to track phospholipid biosynthesis in 2.5 mg of control and glaucomatous TM tissues. The enzymes are italicized: *CK*, *Choline kinase*; *CCTCTP*, *Phosphocholine cytidylyltransferase*; *CPT*, *Cholinephosphottransferase*; *PSS1&2*, *Phosphatidylserine synthase 1 &2*; *PEMT*, *Phosphatidylethanolamine N‐ methyltransferase*; *EPT*, *Ethanolamine‐phosphotransferase*; *PSD*, *Phosphoserine decarboxylase*; *ECTCTP*, *Phosphoethanolamine cytidylyltransferase*; *EK*, *Ethanolamine kinase*. (F) ELISA analysis of enzymes. D, E, and F used *n* = 12 control and POAG samples each (6 male and 6 female). The mean ± standard deviation from three independent experiments is shown. *p ≤ 0.02. (G) Representative Western blot from control and glaucomatous Caucasian donors with age and gender (M, Male; F, Female) as indicated. GAPDH is used as a loading control as indicated. (H) Representative PSD enzymatic activity assay in TM and AH from human donors (*n* = 10; total 10 μg protein per sample) using standard procedure. Control and glaucoma are as indicated. Mean ± standard deviation from three independent experiments is shown. The student's t‐test outcome ****p ≤ 0.0001 or ns = not significant is shown. (I) IOP after lentiviral‐mediated PSD overexpression (Genecopoeia Inc.) in normal DBA/2J‐*Gpnmb*+/SjJ and (J) PSD depletion in glaucomatous DBA/2 J mice, respectively. Sample size of *n* = 10 DBA/2 J‐*Gpnmb*+/SjJ and *n* = 20 DBA/2J were used per group for experiments described in I and J respectively, ANOVA was used to determine significance. Mean ± SD, ****p ≤ 0.0001 difference compared to controls. (K) Representative Western blot analyses of TM from mice using an anti‐PSD rabbit polyclonal antibody (Abcam Inc.). The Coomassie blue stained gel shows equal protein loading. Unless stated otherwise, the asterisk denotes student's *t*‐test outcome.

Our analysis showed total TM tissue lipids undergoing a significant decrease in glaucoma compared to controls (Figure 1A). Consistent with total lipids, total PC level is also decreased in glaucomatous TM (Figure 1A), commensurate with a slightly increased PC level in the AH (Figure 1B). In contrast, our analyses demonstrated a different pattern for other (non‐PC) major membrane phospholipids; that is, decreased PS and increased PE levels in both human glaucomatous AH and TM (Figure 1A,B). We explored whether potential biosynthetic pathway aberrations (Figure 1C) may contribute to observed PS and PE patterns in glaucoma.

The steady‐state levels of precursors feeding the biosynthetic pathway were all within the normal range in glaucoma (Figure 1D). Independent analytical approaches (immunoreactivity and Western blot analysis, Figure 1E–G) revealed that the interconversion enzyme PSD levels were significantly elevated, and its activity was altered in human glaucomatous TM (Figure 1E,H).

We next utilized two model mouse systems, a normal mouse DBA/2J‐*Gpnmb*+/SjJ and a genetically matched glaucomatous DBA/2J mice to test the role of PSD in IOP elevation. PSD enzyme overexpression in the TM of normal DBA/2J‐*Gpnmb* ^+^/SjJ mice was sufficient to elevate IOP over 12 days, while depleting PSD expression in the TM of matched glaucomatous DBA/2J mice reduced it (Figure 1I,J). We verified overexpression and downregulation of PSD in both mice respectively (Figure 1K). Thus, altered PSD levels and activity, as well as concomitant changes in PS and PE, may contribute to IOP elevation in glaucoma patients.

### 3.2 Phospholipid Species Level Differences Suggest Altered Lipid Transport in Glaucoma

**FIGURE 2:**
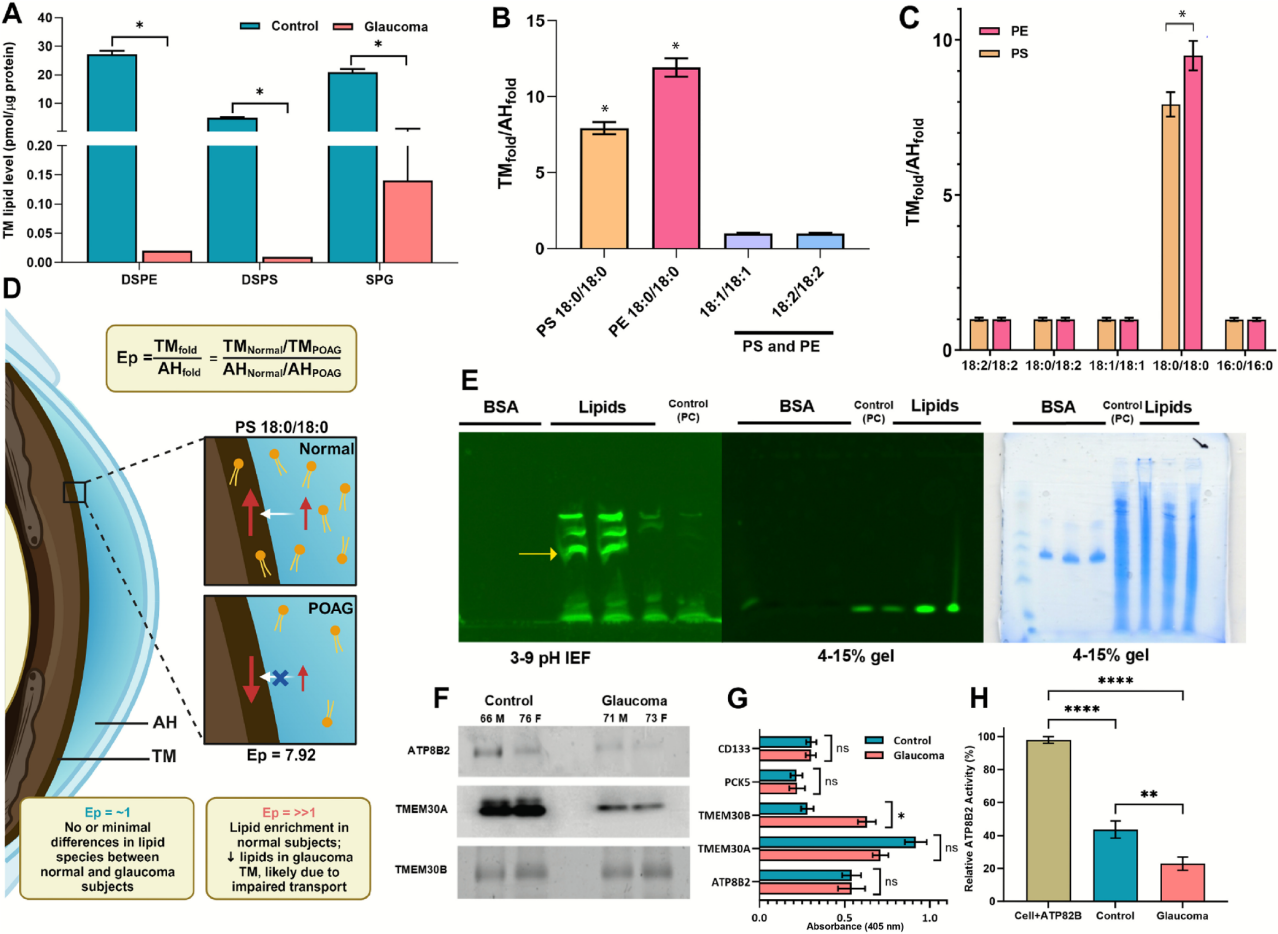
Glaucomatous TM demonstrates altered phospholipid transport. (A) Differences in levels of DSPE, DSPS, and SPG between normal and POAG TM. (B) The TM_fold_/AH_fold_ (normal to POAG for both TM and AH) ratio, for PS and PE lipids 18:0/18:0. Sample size *n* = 12 each (control and POAG, 6 male, 6 female) was used for experiments in A, B. (C) The TM_fold_/AH_fold_ (normal to POAG for both TM and AH) ratio of synthetic isobaric variants of PS and PE lipids that were introduced to AH in controls and POAG whole globes and evaluated after 2–5 h in TM and AH. Sample size *n* = 6 each (3 female and 3 male) was used for control and POAG groups. (D) An illustration describing the Enrichment parameter (Ep) termed for TM_fold_/AH_fold_. (E) Representative isoelectric focusing gel (pH 3–9) of UV cross‐linked TM proteins with NBD‐PS 18:1/18:1 (left). Same as above run on a 4%–15% (middle) representative Coomassie blue stained 4%–15% (right). The yellow arrow shows a band absent in controls. Proteins found crosslinked were identified from this band. (F) Representative Western blot results of ATP8B2, TMEM30A, TMEM30B. (G) Normalized levels of representative ELISA of ATP8B2, TMEM30A, TMEM30B and interacting protein PCSK5 in normal and POAG TM tissues. CD133 has been used for normalization and as control. POAG had about 30% CD133 level compared to control. The lipid rafts from TM tissue were isolated using two different detergent free methods prior to performing estimations. Results are mean ± standard deviation from *n* = 12 samples in each group (6 male and 6 female). The POAG tissues were found to have significant differences compared to controls. (H) Relative activity of ATP8B2 measured using phosphatidylserine PS (18:0/0:0) m/z [M‐H]^−^ = 524.3 and PS (12:0/12:0) or 1,2‐didodecanoyl‐sn‐glycero‐3‐phosphoserine m/z [M‐H]^−^ = 622.3 in a mass spectrometric assay. The activity was validated using CRISPR‐Cas9 depletion of ATP8B2 in primary TM cells (negative control, not shown). About 10 mg of TM tissue for each (control, glaucoma, *n* = 10 each, 5 male, 5 female) was used for a standard assay. Accumulation of product has been represented as percent activity in comparison to 10^8^ primary TM cells overexpressing ATP8B2 at 80% confluence, taken as ~100% (indicated as cell + recombinant or rATP8B2). Unless stated otherwise, the asterisk denotes student's *t*‐test outcome.

We conducted an extensive profiling of various lipid classes from the AH and TM obtained from subjects with healthy eyes and those with POAG or hypertensive conditions, both in humans and mice respectively (Metabolomics Workbench Study IDs: ST0000579, ST0000580, ST0000581, ST0000582, ST0000612, ST0000613, ST0000616, ST0000620, ST0000624). Our investigation revealed significant differences in the levels of closely related lipid species within the TM of healthy compared to POAG samples when analyzed with high‐resolution instruments (Figure S1) in line with what was found with moderate‐resolution instruments earlier [31]. Specifically, we observed significantly reduced levels of DSPE (PE 18:0/18:0; PubChem Compound ID 447078), DSPS (PS18:0/18:0; PubChem Compound ID 9547096), and SPG (C17Sphinganine‐1‐phosphate; PubChem Compound ID 3247041) in human POAG compared to control (Table 1; Figure 2A).

**TABLE 1:** Endogenous aqueous humor (AH) lipids enriched in normal.

m/z	Description	Serial #
750.4638	PE(18:0/18:0) (1,2‐dioctadecanoyl‐sn‐glycero‐3‐phosphoethanolamine)	1
791.5676	PS(18:0/18:0)	2
547.60	1‐octadecanoyl‐sn‐glycero‐3‐phospho‐L‐serine (sodium salt) (18:0) Lyso‐PS	3
810.03	1,2‐dioleoyl‐sn‐glycero‐3‐phospho‐L‐serine (sodium salt) 18:1 PS	4
568.7258	Cer (d18:0/18:1(9Z))	5
566.00	C18:1 Dihydroceramide (d18:0/18:1(9Z)) (N‐oleoyl‐D‐erythro‐sphinganine)	6
679.2484	Cer (d18:1/26:1(17Z))	7
804.5194	SM (d16:1/25:0)	8
303.4092	C17 Sphinganine	9
367.2488	C17 Sphinganine‐1‐phosphate	10
649.90	1,2‐ditridecanoyl‐sn‐glycero‐3‐phosphocholine PC(13:0/13:0)	Control

Closely related PE and PS species (18:1/18:1 and 18:2/18:2), as well as those with minor variations in acyl chain lengths (16:0/16:0 and 20:0/20:0), exhibited distinct trends in TM (Figure S2A,B) and AH compared to DSPE or DSPS (18:0/18:0).

We examined the fold change ratio of lipids with minor differences (acyl chain length or unsaturation) in TM to AH between control and POAG subjects (Figure 2B). We performed experiments with synthetic lipids of various acyl chain lengths and normal as well as POAG human globes to evaluate whether lipid partitioning from AH to TM or transport is different for lipid species with closely related acyl chain lengths or variation in unsaturation (Figure 2C). We introduced the term enrichment parameter (Ep) to describe partitioning of a lipid from AH to TM or transport leading to its greater distribution or enrichment in TM (Figure 2D).

Through crosslinking experiments involving fluorescent analogs of DSPS lipids and mass spectrometry, we identified flippase ATPase phospholipid transporting 8B2 (ATP8B2) as the protein involved with DSPS lipid transport (Figure 2E). For this purpose, we used either NBD‐PC (control) without TM extract or NBD‐PC with TM extract and NBD‐DSPS or NBD‐PC lipids with TM extract on a 3–9 pH isoelectric focusing gel (IEF), which separated additional fluorescent bands (yellow arrow), from where ATP8B2 was consistently identified by mass spectrometry with high confidence (Figure 2E). In contrast to IEF gels, 4%–15% gradient gels did not separate these additional crosslinked bands compared to controls. Usage of bovine serum albumin (BSA) in place of TM extract as control did not result in crosslinking with DSPS, indicating specific binding with proteins in TM extract. These experiments consistently identified ATP8B2 when isobaric analog of DSPS was used in place of fluorescent analog of DSPS. Our further follow‐up revealed decreased ATP8B2 in POAG (Figure 2F). Both ATP8B2 and transmembrane protein 30A (TMEM30A) were found to decrease in glaucoma compared to control (Figure 2F). However, when we normalized the samples using CD133, which also served as control, we did not observe statistically significant differences in ATP8B2 and TMEM30A levels between glaucoma and control (Figure 2G). Transmembrane protein 30B (TMEM30B), on the other hand, was significantly elevated then. Of note, POAG had about a 30% CD133 level compared to control. Despite the similarity in ATP8B2 levels between control and glaucoma samples after normalization, its activity was found to be significantly reduced when compared to the control group (Figure 2H).

### 3.3 PSD Directly Modulates IOP While ATP8B2 Potentially Amplifies Its Effect

**FIGURE 3:**
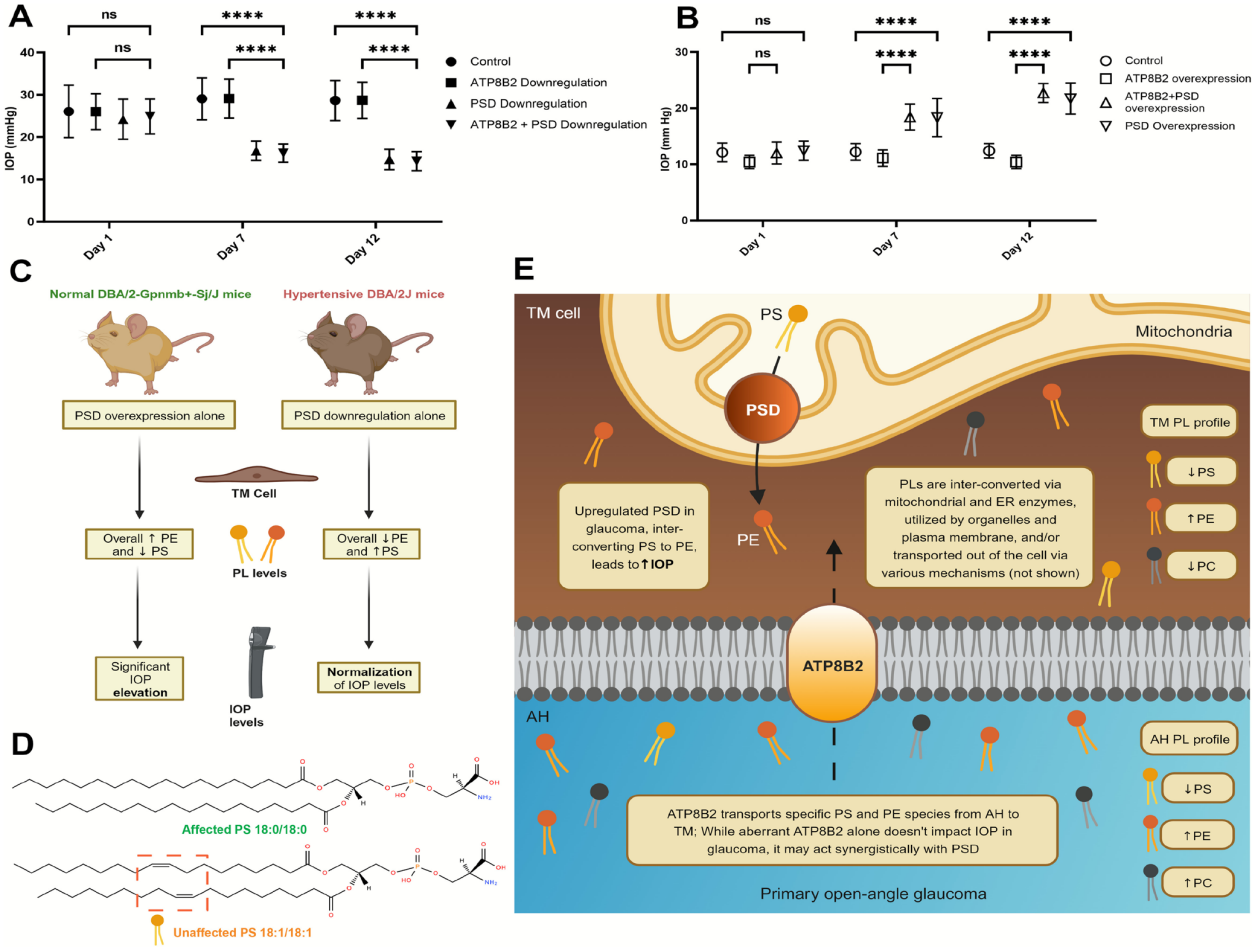
PSD manipulation directly impacts IOP, while ATP8B2 appears to have a limited effect. (A) IOP effects of downregulation of PSD alone, ATP8B2 alone, and PSD combined with ATP8B2 in hypertensive DBA/2J mice (*n* = 20/group) on Days 1, 7, and 12 compared to control, (B) IOP effects of overexpression of PSD alone, ATP8B2 alone, and PSD combined with ATP8B2 in normotensive DBA/2‐Gpnmb+‐Sj/J mice (*n* = 10/group) on days 1, 7, and 12. ANOVA was performed to determine significance for experiments in A and B, ****p ≤ 0.0001. (C) PSD modulation in mice alters PE and PS levels in TM and AH, and directly impacts IOP. (D) Reduced ATP8B2 activity in glaucoma leads to altered transport of specific PLs with minor structural variations (affected) compared to closely related species within the same PL class (unaffected). (E) Upregulated PSD leads to ocular hypertension in POAG, while ATP8B2 does not appear to exert a significant impact on IOP, though it may amplify PSD effects. *ER*, *Endoplasmic reticulum*.

We evaluated the impact of PSD and ATP8B2, both alone and in combination, on IOP. In glaucomatous DBA/2J mice, the downregulation of PSD, as well as the simultaneous downregulation of PSD and ATP8B2, resulted in a substantial reduction in IOP on days 7 and 12 in these hypertensive mouse models (Figure 3A). In contrast, when ATP8B2 alone was downregulated, it did not exert a significant influence on IOP over time, leading to IOP levels comparable to those of the control group (Figure 3A). Furthermore, in normotensive DBA/2‐Gpnmb+‐Sj/J mice, the overexpression of PSD and the combined overexpression of ATP8B2 and PSD significantly elevated IOP levels when compared to the control group (Figure 3B). Conversely, the overexpression of ATP8B2 alone did not produce a substantial impact on IOP (Figure 3B).

## 4 Discussion

Cellular outer boundaries are rich in phospholipids and act as transducers, detecting alterations in the external environment. The TM cell membrane components directly sense pressure changes and participate in the regulation of IOP homeostasis. Specific phospholipid alterations have been identified in the TM and AH in glaucoma [23, 24].

Our analysis of TM showed a decrease in total lipids in glaucoma compared to controls (Figure 1A). We found that the phospholipid PC is significantly elevated in glaucomatous AH, while depleted in the TM (Figure 1A,B). This finding is consistent with pathological membrane disintegration, which leads to the spillage of the cell's most abundant phospholipid [4, 5] into the AH bathing it. Overall, the decreased TM total lipid and decreased total TM PCs are likely attributable to TM cellular degradation as a result of glaucomatous changes to the anterior segment microenvironment [23] and are consistent with increased PC presence in the AH.

In contrast, glaucomatous TM and AH demonstrate lower levels of PS and higher levels of PE (Figure 1A,B) compared to controls. These trends cannot be explained by membrane disintegration and secretion of PLs into AH. These findings of PS and PE are inconsistent with the logic for total lipids and total PCs. Therefore, we evaluated the enzymes involved in phospholipid interconversion (Figure 1C). We note that the steady state values of metabolite inputs lacked demonstration of any potentially applicable discernable difference (Figure 1C–E), strongly suggesting an aberration in the interconversion step. Our analysis showed a discernable increase in PSD levels and activity in glaucoma compared to controls (Figure 1E–H). PSD is a ubiquitous enzyme preferentially located at the inner mitochondrial membrane [36] and produces PE by PS decarboxylation, explaining the concomitant higher levels of PE and lower levels of PS observed in glaucoma. We next evaluated live animal models for the potential role of PSD in IOP elevation. We found that overexpressing PSD in normotensive DBA/2‐Gpnmb+‐Sj/J mice is sufficient to elevate IOP (Figure 1I), while depleting PSD in hypertensive DBA/2 J mice is enough to lower it to a normal range (Figure 1J).

Lipid synthesis and interconversions take place intracellularly. Consequently, if only aberrant PSD is implicated, lipid species within the same class would exhibit a degree of uniformity. Further, since AH largely lacks enzymes, no changes in PLs are expected to occur once they are released from anterior segment structures into the AH. As shown in Figure 1A, as a lipid class total PS lipid is decreased in POAG, while total PE class of lipids is increased in POAG both in TM and AH. While the class trend shows a decrease for PS and increase for PE, individual lipids differ in degrees of change and some even do show an opposite trend. We had noticed this large fold changes (1–2‐fold for most species compared to > 10 000‐fold for other species in TM or AH) in our previous profiling with moderate resolution instruments [31]. We found two closely related species such as PS 16:0/16:0 and PS 18:0/18:0 differ immensely when fold changes between control and POAG are very different (Figure S2A,B). For many PS and PE species, these fold changes are ≤ 1.0, that is distribution is similar between AH and TM (Figure S2B). However, for many, the TM concentration appears depleted compared to POAG even when these species are present in AH. Parsing through all profiling data regardless of whether moderate or high resolution confirmed this trend. This data suggested a difference in partitioning from AH to TM in closely related phospholipids, such as PS 18:0/18:0 but not PS 18:1/18:1 or PS 16:0/16:0, led us to investigate a potential aberration in lipid transport in POAG TM. It is important to emphasize that synthesis aberrations would typically lead to uniform changes across different lipid species within their respective classes and would not manifest as simultaneous differences in both TM and AH. Despite the overall changes in PE and PS lipid classes we described (Figure 1A), the levels of several individual species within those classes did not follow the general trend. Compared to other species, DSPS or DSPE have only slight differences in acyl chain length, degree of unsaturation, or the presence of double bonds (Figure S2A,B). Within TM, the differences observed may be attributed to synthesis and interconversion alterations. However, the simultaneous decrease of these species (PS, PE) in both TM and AH (Figure 1A) can be explained only if an additional component, such as transport, is impaired. Among the lipids assessed, several displayed a TM_fold_ to AH_fold_ ratio, or Ep, of or near 1.0, suggesting minimal variation between normal and diseased samples. Conversely, several lipids, such as DSPE, DSPS, and SPG, had an Ep between 8 and 10. A larger Ep indicates lipid enrichment in healthy TM compared to glaucomatous. We employed the following reasoning: a numerator (TM fold change between control and glaucoma) near equal to the denominator (AH fold) suggests that the lipid levels at the TM in POAG are identical to the normal TM tissue. The higher Ep, on the other hand, suggests lipid enrichment in normal eyes but likely a lack of transport from AH to TM in POAG. The Ep for PS and PE with acyl chain length 16:0/16:0 or 18:1/18:1 was close to 1.0, whereas the Ep for 18:0/18:0 was 8–10 (Figure 2B). To corroborate our Ep findings from lipid profiling, we introduced five isobaric synthetic lipid species into human whole globe organ cultures from control and glaucomatous subjects (Figure 2C). Our results consistently demonstrated DSPS or DSPE enrichment in the TM of controls but not in POAG. We evaluated the enrichment parameter, Ep, between glaucoma and controls, for individual species across various PL classes (Figure 2D). Ep close to 1 indicates no or minimal changes in these species between glaucoma and control. On the other hand, the larger the Ep, the higher the lipid enrichment in normal subjects compared to those with glaucoma. An elevated Ep also implies that factors other than synthesis, such as transport mechanisms, may be impaired. Upon our investigation, we anticipated finding similar or identical fold changes for all species within the same lipid class. However, we noticed substantial variations among different PS and PE species that vary by only a few acyl‐chain lengths or unsaturation (Figure 2B,C). Examining our previously published quantitative proteomics/proteomics data [30, 37], we identified ATP8B2 as a compelling candidate to account for the observations in our lipidomics results. Subsequent crosslinking and activity assays revealed reduced ATP8B2 levels in glaucomatous TM compared to controls (Figure 2F,G). ATP8B2 is a ubiquitous flippase, particularly abundant in the central nervous system [38]. This type of ATPase has been implicated in a wide variety of processes, including cell volume regulation, nerve cell excitability, substrate uptake, and signaling pathways [39]. In the anterior segment, ATP8B2 transports phospholipids inwards from AH to the TM.

We analyzed ATP8B2 subunit levels and/or interactors in non‐reducing gels with established capillary electrophoresis methods [40]. Our data showed reduced ATP8B2 activity in POAG (Figure 2H) but also altered levels of the transmembrane proteins TMEM30A and TMEM30B (Figure 2E–G). These findings are consistent with differences in fold changes in lipids species compared to other closely related species of similar structure in glaucoma. The current short‐term in vivo modulation does not suggest that ATP8B2 alone, which transports specific PL species with minor structural variations (Figure 3D), is sufficient to result in demonstratable alteration in IOP (Figure 3B). This may hold true for longer‐term reductions and warrants further investigation.

Sterols have been shown to alter IOP, which acts on sterol regulatory element‐binding proteins (SREBPs), a key transcription factor in lipid regulation. While modulation of SREBP and its cleavage‐activating protein (SCAP) affects cholesterol levels, however, the most pronounced alterations of this modulation are phospholipid profiles [19] underscoring the importance of phospholipid alterations in membrane mechanosensing and IOP regulation.

In summary, our study sheds light on two distinct mechanisms contributing to the differences in PL profiles between POAG eyes and healthy ones (Figure 3E). The first mechanism involves the upregulation of PSD, resulting in an excessive interconversion of PE from PS. The second mechanism centers around an aberrant transport of PLs mediated by ATP8B2, leading to significant variations among closely related species within the PE and PS classes. Notably, our findings suggest that downregulating PSD alone is sufficient to reduce IOP, while ATP8B2 does not appear to exert a significant impact on IOP, though it may amplify PSD effects.

## Author Contributions

Genea Edwards: methodology, investigation, writing – review and editing, visualization; Ruminder Kaur: visualization, writing – review and editing; Anna Mueller: visualization, writing – review and editing; Noël Ziebarth: methodology, investigation; Richard K. Lee: project administration, funding acquisition, supervision; Sanjoy K. Bhattacharya: conceptualization, methodology, investigation, writing – original draft, writing – review and editing, visualization, project administration, funding acquisition, supervision.

## Funding

National Institutes of Health grant EY016112 (SKB); National Institutes of Health grant EY031292 (SKB); National Institutes of Health grant P30EY014801 (SKB); U.S. Department of Defense grant W81XWH‐15‐1‐0079 (SKB); W.H. Coulter Center grant (SKB); Research to Prevent Blindness (SKB, RKL).

## Disclosure

The authors have nothing to report.

## Conflicts of Interest

None. Richard Lee and Sanjoy Bhattacharya are co‐inventors on a US patent number 10046001 issued on August 14, 2018, Title: Compositions and methods for reducing intraocular pressure. The patent covers lipids including phospholipids as modulators of intraocular pressure. However, the patent is not congruent with work described here.

## Supporting information


**Appendix S1:** fsb271451‐sup‐0001‐AppendixS1.doc.

**Figure S1:** Representative confirmation of lipids via fragment ion signatures and differences in phospholipid transport in the POAG TM.
**Figure S2:** Vast difference in fold change in several similar phospholipids in the TM in POAG.
**Figure S3:** PSD level compared to beta actin.
**Figure S4:** Probing the ATP8B2 blots in Figure 2 with anti‐CD133 and anti‐beta actin.
**Figure S5:**. Probing Figure 3 animal model proteins for ATP8B2 and PSD.

## Data Availability

Stored in repository.
