# Supplementary material for: Phospholipid Interconversion and Transport Are Altered in Glaucoma
Source: FASEB J. 2026 Jan 20;40(2):e71451. doi: 10.1096/fj.202504068R (PMC12817104; doi:10.1096/fj.202504068R)
Supplement: Supplementary file 1 — Appendix S1: fsb271451‐sup‐0001‐AppendixS1.doc. [file FSB2-40-e71451-s002.doc]

**Supplemental Methods**

**Analyses of trabecular meshwork (TM) and aqueous humor (AH) lipidome**

*Human TM(Bhattacharya et al., 2005) or mouse TM(Goel et al., 2012) was dissected out of the enucleated eyes under a dissecting microscope by a trained ophthalmologist following published procedures practiced by our group.* Samples from patients with glaucoma were included in the study if death to enucleation was less than 24 hours for all experiments (except for Western blot analysis, where this criterion was relaxed for up to 32 hours, based on our prior experimental experience) and for at least some details of static perimetry data was available indicating progressive visual field loss. The availability of some general medical history was also considered for inclusion. The exclusion criteria for patients were the presence of any other eye disease, especially the record of any other retinal diseases. Refractive error-related issues or cataract was not part of the exclusion criteria. The experiments used mice of both genders. Any mice that developed excessive hyperemia, prolapse, tumor was excluded from the study. Mice with other conditions of concern were excluded from the study if recommended by attending veterinarian. Based on initial statistical analysis we have contingenies for natural attrition by unwarranted natural death. All studies were subjected to inclusion of randomization in their study design. All presented results are derived from at least three independent biological replicate experiments.

*Lipid extraction*

Lipids were extracted using Bligh & Dyer method(Bligh and Dyer, 1959). Protein content in sample aliquots was quantified by Bradford protein assay(Bradford, 1976) or PhastGel format(Amelinckx et al., 2009) with densitometry for normalization purposes.

*Moderate resolution mass spectrometry*

For these measurements suitable modification of published(Bhattacharya, 2013; Shaner et al., 2009; Yang et al., 2009) papers were used. Briefly, we used TSQ Quantum Access Max (Thermo Fisher Scientific) triple quadrupole mass spectrometer driven by Xcalibur 2.3 software (Thermo Fisher Scientific). Samples were injected as direct infusion using a Triversa Nanomate driven by Chipsoft 8.3 (Advion Inc.). The samples were run with and without addition of lipid standards for each class of lipids analyzed as done in our previous studies(Aljohani et al., 2014; Aljohani et al., 2013; Aribindi et al., 2013; Edwards et al., 2014a; Edwards et al., 2014b; Guerra et al., 2014; Guerra et al., 2015). DBA/2J aqueous humor phospholipids was profiled and reported previously(Wang et al., 2015). We have deposited both previously reported AH(Wang et al., 2015) and new DBA/2J TM profiling data at Metabolomics workbench: ST000579-ST000582. The previously reported AH and TM Sphingolipid profiling data(Guerra et al., 2014) has been deposited as well: ST000612-ST000613. A non-mammalian/synthetic lipid standard was used during extraction.

*High resolution mass spectrometry*

Lipid samples were loaded onto a HPLC Ascentis® Express C18 Column (15 cm, 2.1 mm, 2.7 µm) with a C18 (octadecyl) phase as matrix active group on a fused-core particle platform and 90 Å pore size. For reverse-phase chromatography, an HPLC Thermo Scientific Accela LC System instrument was used to elute the lipids onto Q Exactive mass spectrometer (Thermo Fisher Scientific). The lipid samples were kept at -80ºC until use and then resuspended in chloroform:methanol (1:1, v/v).

High performance liquid chromatography (HPLC): Reverse-phase chromatographic separation was achieved with the Accela Autosampler, Accela 600 pump and Acclaim C30 column: 3 µm, 2.1x150 mm (Thermo Fisher Scientific, Waltham, MA). The column temperature was maintained at 30 ºC (negative mode) or 45ºC (positive mode) and tray temperature at 20ºC. The mobile phase consisted of solvent A (60:40 CH3OH: H2O with 0.2% FA and 10 mM NH4CH3CO2) and solvent B (60:40 CH3OH: CHCl3 with 0.2% FA and 10 mM NH4CH3CO2), with a constant flow rate of 260 μL/min. All solvents are LC/MS grade. A starting mobile phase composition of 35% B was maintained for 13 minutes, increasing linearly to 100% B over 0.8 minutes, returning to initial conditions in 0.7 minutes, and decreasing to 0% B for 2 minutes, for a total elution time of 20 minutes. The loop size and injection volume were 5 μL.

Mass spectrometry: The Q Exactive mass spectrometer operated with heated electrospray ionization (HESI) in positive and negative modes separately for each lipid sample. The spray voltage was set to 4.4 kV. The heated capillary was maintained at 310ºC (negative mode) or 350ºC (positive mode) and heater at 275°C (positive mode). The S-lens radio frequency (RF) level was set to 70. The sheath gas flow rate was set to 30 (negative mode) or 45 units (positive mode) and auxiliary gas to 14 (negative mode) or 15 units (positive mode). External mass calibration in positive and negative mode was performed with the standard calibration mixture every 7 days (or earlier than 7 days). Full scan (m/z 150-1500) used resolution 70,000 at m/z 200 with automatic gain control (AGC) target of 1x10^6^ ions and maximum ion injection time (IT) of 100 ms. Data-dependent MS/MS (top10) were acquired with the following parameters: resolution 17,500; AGC 2x10^5^ (negative mode) or 1x10^5^ (positive mode); maximum IT 100 ms (negative mode) or 75 ms (positive mode); 1.3 m/z isolation window; underfill ratio 0.1%; intensity threshold 2x10^3^ (negative mode) or 1x10^3^ (positive mode); dynamic exclusion time 3 s (negative mode) or 10 s (positive mode). Normalized collision energy (NCE) settings were 40± 25% for negative mode and 30, parallel with 19± 5% for positive mode. We had used ratiometric quantification using internal standards for phospholipids and sphingolipids (same as in our previous studies(Aribindi et al., 2013; Edwards et al., 2014a) but in 2-100 pmol range). The data was obtained with and without addition of internal standards to facilitate ratiometric quantification. We also performed relative quantification between control and glaucomatous group to judge relative levels from without internal standard added datasets.

*Data Analytics*

The analyses were performed using LipidSearch 4.1 (Thermo Fisher Scientific) and MZmine 2.9 with LIPID MAPS derived database. Quantification utilized class-specific standards in a 2-step process developed for automated lipid quantification(Enriquez-Algeciras and Bhattacharya, 2013; Yang et al., 2009). These concentrations were normalized to total protein content.

Lipid identification and relative quantification: Lipid identification and relative quantification utilized LipidSearch 4.1 software. The search criteria were as follows: product search; parent m/z tolerance 5 ppm; product m/z tolerance 10 ppm; product ion intensity threshold 1%; filters: toprank, main isomer peak, FA priority; quantification: m/z tolerance 5 ppm, retention time tolerance 1 min. The following adducts were allowed in positive mode: +H, +NH4, +H-H2O, +H-2H2O, +2H and negative mode: -H, +HCOO, +CH3COO, -2H. All classes were selected for search.

Data processing: Positive and negative mode identifications of samples were aligned in LipidSearch 4.1, allowing calculation of unassigned peaks. The following settings were used: product search; alignment method max; retention time tolerance 0.1 min; filters: toprank, main isomer peak; M-score 5; molecular lipid identification grade: A-B (A: lipid class and fatty acid completely identified or B: lipid class and some fatty acid identified). Peaks with the same annotated lipid species were merged.

**Enzyme activity**

The enzymatic activities for phospholipid conversion pathways were determined from 10 µg of total protein extract of control (n=10) and glaucomatous TM tissue (n=10) utilizing mass spectrometric analysis for the formation of product or depletion of substrate (see below) or separated by thin-layer chromatography followed by counts for radioactivity.

For mass spectrometry analyses, a 100 µL final reaction volume was started by the addition of protein extract and incubated at 37ºC for 2 h. Reaction was sampled at 0 min, 30 min, 1 h and 2 h by transferring 10 µL of sample aliquot into 200 µL of chloroform:methanol mixture (1:1, v/v) with 10 µM BHT and vortexed. Lipid extraction was performed using Bligh&Dyer method(Bligh and Dyer, 1959). Mass spectrometry analyses were performed on Q Exactive mass spectrometer. Quantification utilized class specific lipid standards (Avanti Polar Lipids Inc.).

For radioactivity studies, a 100 µL final reaction volume was started by the addition of protein extract and incubated at 37ºC for 20 min. Reaction was stopped by adding 200 µL of chloroform: methanol (1: 1, v/v) with 10 µM BHT and vortexed. Lipid extraction was performed as described above. Labeled product was separated by thin-layer chromatographically and counted for radioactivity.

| **Enzyme** | **MS analysis** | | | **Radioactivity assay** |
| --- | --- | --- | --- | --- |
|  | Reaction mixture | Substrate [m/z] | Product [m/z] | Reaction mixture |
| **choline kinase (CK)** | 0.2 M Tris-HCl (pH 8.75), 100 mM Mg(CH_3_COO)_2_, 100 mM ATP, 5 mM choline chloride | 140.63 | 184.15 | 0.2 M Tris-HCl (pH 8.75), 100 mM Mg(CH_3_COO)_2_, 100 mM ATP, 5 mM [Me-^14^C]choline chloride (0.7 Ci/mol) |
| **phosphocholine cytidylyltransferase (CCTCTP)** | 20 mM Tris-malate (pH 6.0), 15 mM Mg(CH_3_COO)_2_, 4.2 mM CTP, 1.6 mM phosphorylcholine | 184.15 | 489.33 | 20 mM Tris-malate (pH 6.0), 12 mM Mg(CH_3_COO)_2_, 4.2 mM CTP, 1.6mM [Me-^14^C] phosphorylcholine (1000 cpm/µmol) |
| **choline phosphotransferase (CPT)** | 4 mM glutathione, 10 mM MgCl_2_, 34 mM Tris-HCl (pH 7.4), 0.08 mM CDP-choline, 1.0 mg/mL diglyceride, 0.01 mg/mL Tween 20 | 489.33 | 782.57 | 4 mM glutathione, 10 mM MgCl_2_, 34 mM Tris-HCl (pH 7.4), 1.0 mg/mL diglyceride, 0.01 mg/mL Tween 20, 0.08 mM CDP-[Me-14C]choline (0.02 µCi) |
| **phosphatidylserine synthase 1 (PSS1)** | 50 mM Tris-HCl (pH 8.0), 0.6 mM MnCl_2_, 0.5 mM L-serine, 0.2 mM CDP-diacylglycerol, 4 mM Triton X-100 | 782.04 | 782.49 | 50 mM Tris-HCl (pH 8.0), 0.6 mM MnCl_2_, 0.5 mM L-serine, 0.2 mM CDP-diacylglycerol, 4 mM Triton X-100, L-[^3^H]serine (10,000 cpm/nmol) |
| **phosphatidylserine synthase 2 (PSS2)** | 100 mM CaCl_2_, 40 mM hydroxylamine, 20 mM HEPES (pH 7.4), 0.4 mM L-serine, 0.2 mM ethanolamine | 740.52 | 782.49 | 100 mM CaCl_2_, 40 mM hydroxylamine, 20 mM HEPES (pH 7.4), [^14^ C]serine solution (151 µCi/µmol), or [^14^C]ethanolamine solution (55 µCi/µmol). |
| **phosphatidylethanolamine N-methyltransferase (PEMT)** | 125 mM Tris-HCl (pH 9.2), 5 mM DTT, 1 mM Triton X-100, 2 mM PE, 0.4 mM PMME | 740.52 | 782.57 | 125 mM Tris-HCl (pH 9.2), 5 mM DTT, 1 mM Triton X-100, 2 mM PE, 0.4 mM PMME and [Me-^3^H]AdoMet (21 mCi/mmol) |
| **ethanolamine-phosphotransferase (EPT)** | 50 mM Tris-HCl (pH 8.0), 20 mM MgCl_2_, 20% glycerol, 10 mol% diradylglycerol, 0.4 mM CDP-ethanolamine | 447.25 | 740.52 | 50 mM Tris-HCl (pH 8.2), 3 mM diacylglycerol, 10 mM MnCl_2_, 2 mM NADH, 30 µM CDP-[1,2-^14^C]ethanolamine (25 Ci/mol), 1.5% Thesit |
| **phosphatidylserine decarboxylase (PSD)** | 50 mM imidazole-HCl (pH 7.0), 6 mM phosphatidylserine, 36 mM Triton X-100 | 782.49 | 740.52 | 0.2 M KH_2_PO_4_ (pH 7.0), 0.2% Triton X-100, 0.1 to 0.2 mM phosphatidyl-[1-^14^C]serine (100,000 cpm/mol) |
| **phosphoethanolamine cytidylyltransferase (ECTCTP)** | 40 mM Tris (pH 7.8), 20 mM MgCl_2_, 100 mM DTT, 40 mM CTP (pH 7.0), 10 mM phosphoethanolamine | 142.07 | 447.25 | 40 mM Tris (pH 7.8), 20 mM MgCl_2_, 100 mM DTT, 40 mM CTP (pH 7.0), 10 mM phospho[1,2-^14^C]ethanolamine (0.5 mCi/mmol) |
| **ethanolamine kinase (EK)** | 100 mM MOPS (pH 7.8), 6 mM MgCl_2_, 5 mM ATP, 2 mM ethanolamine | 62.09 | 142.07 | 0.3 M glycylglycine, sodium salt, (pH 8.5), 100 mM disodium ATP, 100 mM MgCl_2_, 5 mM [1,2-^14^C]ethanolamine-HCl (1 Ci/mol) |
| **Substates**: choline chloride, choline phosphate, CDP choline, PC(18:2(9Z,12Z)/18:2(9Z,12Z)), PE(18:2(9Z,12Z)/18:2(9Z,12Z)), PE(18:2(9Z,12Z)/18:2(9Z,12Z)), CDP ethanolamine, PS(18:2(9Z,12Z)/18:2(9Z,12Z)), etholamine phosphate and ethanolamine respectively. **Products**: choline phosphate, CDP choline, PC(18:2(9Z,12Z)/18:2(9Z,12Z)), PS(18:2(9Z,12Z)/18:2(9Z,12Z)), PS(18:2(9Z,12Z)/18:2(9Z,12Z)), PC(18:2(9Z,12Z)/18:2(9Z,12Z)), PE(18:2(9Z,12Z)/18:2(9Z,12Z)), PE(18:2(9Z,12Z)/18:2(9Z,12Z)), CDP ethanolamine and ethanolamine phosphate respectively. | | | | |
|
|
|

**Steady-state level measurements**

Steady-state analyses of radiolabeled phosphatidylcholine and phosphatidylethanolamine precursors for control (n=10) and glaucomatous TM tissue (n=10) were determined by thin-layer chromatography followed by counts for radioactivity of individual lipids. Standard buffer mixture of 10 mM Tris-succinate pH 7.0, 12 mM magnesium acetate, tracer quantities of radioactive choline or ethanolamine-containing compounds and 5 µg of protein extract in a reaction volume of 100 µL was utilized. End-product precursors: choline, choline phosphate, CDP-choline, CDP-ethanolamine, ethanolamine phosphate and ethanolamine were extracted using Bligh & Dyer extraction method(Bligh and Dyer, 1959) then separated by chromatography and measured by radioactivity.

**RNA extraction and reverse transcription PCR**

RNA was isolated from TM nuclear extract and AH using the miniRNA extraction kit (Stratagene Inc.) according to manufacturer’s protocol. Total RNA was dissolved in DEPC-treated distilled water and converted to cDNA with the oligo(dT) 12-18 (Invitrogen Inc.). For detection of Stt3b mRNA, RT-PCR of TM or AH-derived mRNA was carried out with primers (for Stt3b: Forward GGAGGGGTTATTGGCTATTCT, Reverse ACAAGCCAGTGTTCTGATGTA).

**TM cell culture**

The primary TM cells derived from 7-15 years old cadaveric eyes (all Caucasian and male donors) not subjected to head or ocular trauma displayed regular filopodia retraction and or expansion. TM cells were isolated from corneal donor tissues and cultured using established protocols(Stamer et al., 1995).The microscopy was performed following established methods(Goel et al., 2011). Cells were authenticated following established methods.

**Western blot and ELISA experiments**

Western blot, dot blot and ELISA analyses utilized established protocols(Bhattacharya et al., 2005; Goel et al., 2012). Proteins were separated (10 µg proteins per lane for human TM/other ocular tissues and 5 µg proteins per lane for mice TM/other ocular tissues respectively unless stated otherwise) on a 4-20% SDS-PAGE. Dot blots utilized 1µg protein per spot. Western analyses were performed after transferring the proteins onto a PVDF membrane. The blots were blocked either with 5% BlokHen (Aves Labs Inc.) or 2% BSA in PBS and incubated with antibodies as specified for individual experiments with 0.1% BSA in PBS overnight at 4°C. Incubation was then performed using HRP-conjugated secondary antibodies. Chemiluminescence was used to detect secondary antibodies. For quantitative analyses antibodies coupled to 700/800 nm IR-dyes were used on an Odyssey Infrared Imaging system (Li-Cor Biosciences) as described elsewhere(Bhattacharya et al., 2005). For Western analysis of enzymes, unless stated otherwise protein was extracted from the TM tissues by homogenization in lysis buffer (125 mM Tris-HCl, pH 7.0, 100 mM NaCl, 0.1% Genapol C100, 100 µM BHT).

**Ocular injections**

The mice were anesthetized with intraperitoneal injection of ketamine (100 mg/kg) and xylazine (9 mg/kg). Intravitreal injections were performed under anesthesia using an Ultra Micro Pump II (UMPII; World Precision Instruments Inc.) delivering 0.7 μL viral construct or other agents as appropriate unless stated otherwise. The maximum volume that has been injected in mice eyes in all our studies is 1 µL. A partial-thickness pilot hole was made with a 70 mm long needle to facilitate penetration of the underlying tissue by a fine needle fitted to a 5 μL syringe operable with the UMPII, mounted on a stereotaxic frame. The micropipette was connected to a 5 μL glass syringe (ILS005LT, World Precision Inc.) for delivery. An ointment containing antibiotics was applied to the injection site.

**Overexpression and downregulation of PSD**

The PSD was overexpressed and downregulated in DBA/2J-*Gpnmb*^+^/SjJ and DBA/2J eyes respectively as detailed below.

*Virus construct and confirmation of presence of PSD gene*

The mouse PSD construct (EX-Mm23699-Lv154) was procured from GeneCopeia, Inc. Expression constructs were transformed into DH5α competent cells (Invitrogen). Individual colonies were picked, expanded and expression plasmids were purified with QIAGEN QIAprep Miniprep kit. The presence of gene was verified using PCR (primers: Forward ATGAGGGTCTTCAGCGGAAGCC, Reverse CTCCAGACTCTACCAGACAAGCCA). The PCR amplified bands (570 bp) were excised and extracted from an agarose gel using a QIAGEN Gel Extraction Kit. The PSD gene is expressed under the control of a CMV promoter in this vector. The presence of *PSD* gene in the construct was also confirmed by DNA sequencing.

*Virus production and transfection*

The PSD overexpression construct EX-Mm23699-Lv154 and shRNA against PSD contructs MSH041683-LVRH1GP (set of 4 clones plus one control clone) were transfected separately to HEK293T cells using Lenti-Pac HIV Expression Packaging Kit (GeneCopeia, Inc.; HPK-LvTR-20) using protocol provided by manufacturer. Briefly, 2.5 µg of DNA was incubated with 200 µL Opti-MEM containing 5 µL of Lenti-Pac HIV mix. In a separate tube, 15 µL of EndoFectin Lenti was incubated with 200 µL of Opti-MEM then added to the DNA solution. Mixture was then incubated for 25 min at room temperature then added to the HEK293T cells. Media was changed after 16 h. 48 h post transfection, media containing lentiviral particles was collected, centrifuged and filtrated with 0.45 µm Super Membrane (PALL). The filtrated media was mixed with 100 % PEG (Polyethylene Glycol 6000, USB Corporation) according to 6:4, v:v ratio, and centrifuged for 20 min at 3500 rpm at 4°C. The pellet was precipitated and re-suspended in 300 µL DMEM. 10 µL of PSD lentivirus was mixed with 1 µL of CMV-YFP pLionII lentivirus and the whole mixture was added to HEK293Tcells for transfection. 48 h post transfection, cells were trypsinized and total protein extracted for Western analyses, probing for PSD with corresponding antibody (ab93603, Abcam).

*PSD overexpression and shRNA experiments*

PSD overexpression and shRNA experiments were carried out utilizing 8 months old DBA/2J-*Gpnmb*^+^/SjJ (20 animals: average IOP=9-15 mmHg) and DBA/2J mice (40 animals with average IOP>18 mmHg), respectively. A control scrambled shRNA was used for injection in the eyes in an identical set of control animals to ensure elimination of contralateral eye effects from the results. An equal number of male and female animals were used for these studies to rule out sex as a biological variable. IOP was measured with a TonoLab (Colonial Medical Supply) on anaesthetized mice prior to intracameral injection of collected lentiviral particles (0.7 µL, ~2x 10^8^ transduction unit/mL) using protocols as noted above. The contralateral eye was used as control. A topical anesthetic (tetracaine hydrochloride 0.5%) was applied to the desired eye prior to injection. Subsequent IOP measurements were conducted once daily for 15-21 days. Mice were then euthanized, and TM and AH were collected and proteins extracted for Western analyses probing for PSD.

**Measurement of intraocular pressure (IOP)**

IOP was measured in animals using a rebound tonometer(Chatterjee et al., 2013) (Tonolab, Colonial Medical Supply). Animals were measured when anesthetized by intraperitoneal injection of a ketamine/xylazine as described above. The tonometer was clamped horizontally to a stand to allow perpendicular contact with the central cornea. The tip of the probe was positioned 2-3 mm from the eye. The hand-held rebound tonometer was modified to include a pedal to activate the probe in order to reduce variability. Average IOP was taken from three sets of six measurements for each eye. All measurements were taken 4-7 min after anesthesia(Aihara et al., 2002) and between 11AM and 1PM. A subset of confirmatory IOP measurements were also performed using cannulation methods developed by Dr. Simon John(John et al., 1997; Savinova et al., 2001) on mice usually just prior to end points. Because DBA/2J mice develop calcified corneas, we have used this method on a subset of animals. Anesthetized mice were placed on a heated platform (35-37°C). A drop of BSS (balanced salt solution) was placed on each eye to prevent corneal dehydration. We avoided pressure on the neck that could alter IOP. Eyes of anesthetized animals were cannulated with a very fine fluid-filled glass microneedle. For this purpose, the eye was viewed under a dissecting microscope while the microneedle tip was placed inside a drop of PBS and pressure reading was calibrated to zero. The tip of the microneedle was inserted into the anterior chamber by piercing the cornea over the pupil and using a micromanipulator to place the needle tip 50-100 µm into the chamber. The microneedle was connected to a pressure transducer and a computer system was used to measure and analyze the pressure signal. IOP was recorded at 30 sec intervals for the first two min after ocular entry of microneedle.

**Histology**

Eyes were enucleated (and other tissues if assessed), immediately immersed in 4% paraformaldehyde and incubated for 24 h at 4ºC in dark. After alcohol dehydration, the eyes were embedded in paraffin and the whole globe was mounted and sectioned at 5 µm thick. Hematoxylin and eosin (H&E) staining was performed to assess for any differences in optic nerve integrity and morphology.

**Confocal imaging**

Immunohistochemical and immunocytochemical images were obtained using a confocal microscopy following established protocols(Goel et al., 2011; Goel et al., 2012). Eyes/tissues/cells were fixed with calcium acetate buffered 4% paraformaldehyde unless stated otherwise and stored at 4ºC for 4-24 h. The tissue was excised, embedded into paraffin and sectioned at 10 µm. The cells were placed on the slide. The sections/cells were blocked with 1% BSA in PBS and incubated with antibodies as specified for individual experiments with 0.1% BSA in PBS overnight at 4°C. Next, secondary antibodies conjugated with fluorophores were applied then incubated under dim light for 1 h at room temperature. The sections were washed with PBS and finally rinsed with water. The sections were sealed after application of Vectashield. The images were recorded with a fluorescence microscope (Nikon EFD-3) or on a scanning confocal microscope Leica TCS-SP5 AOBS microscope.

**Perfusion anterior segment culture of human eyes**

Anterior segments of human donor eyes were obtained from the Lions Eye Institute, Miami. The anterior segments of paired donor eyes were set up for *ex vivo* perfusion culture as previously described(Bhattacharya et al., 2009). The Qtracker dyes(Carreon et al., 2016a; Carreon et al., 2016b) were introduced as a bolus into each eye, and then eyes were perfused at a constant flow rate of 2.5 µL/min. Perfusate was collected and anterior segments subjected to analyses.

**Thin-Layer Chromatography (TLC) of Phospholipids**

Phospholipids were separated by thin-layer chromatography (TLC) on silica gel 60 F254 plates (Merck) with minor modifications from published protocols. Lipids were extracted from samples using the Bligh and Dyer method, dried under nitrogen, and dissolved in chloroform: methanol (2:1, v/v). Aliquots (1–5 µL) containing 0.1–2 µg lipid were applied 1 cm from the lower edge of pre-washed plates and developed in chloroform: methanol: acetic acid: water (50:30:8:4, v/v/v/v) to approximately 8 cm from the origin. After air-drying, lipids were visualized by either primuline staining (0.05% in acetone:water, 80:20, v/v) under UV illumination. A subset of confirmatory analysis was also performed by charring following spraying with 10% CuSO₄ in 8% H₃PO₄ and heating at 180 °C for 10 min. Phospholipid species were identified by comparison to authentic standards (phosphatidylcholine, phosphatidylethanolamine, phosphatidylserine, and phosphatidylinositol procured from Avanti Polar Lipids), and relative band intensities were quantified by densitometry using ImageJ software. As needed, data were normalized to an internal standard (dipalmitoyl-phosphatidylcholine) and expressed as relative abundance or mol% of total phospholipid.

**Flippase Activity Assay**

ATP8B2 flippase activity was assessed using a cell-based NBD-lipid uptake and dithionite quenching assay as previously described with minor modifications. Briefly, HEK293T cells transiently expressing human ATP8B2 and its CDC50A subunit were incubated with 2 µM NBD–phosphatidylserine (Avanti Polar Lipids) in Hanks’ balanced salt solution (HBSS) containing 10 mM HEPES (pH 7.4) for 10 min at 25 °C to allow lipid incorporation into the plasma membrane. Cells were washed and treated with 5% fatty-acid-free BSA in HBSS for 5 min to remove surface-accessible lipid, followed by fluorescence quenching with 20 mM freshly prepared sodium dithionite in Tris (pH 9.0) for 5 min on ice to eliminate outer-leaflet fluorescence. Residual fluorescence, representing internalized (flipped) NBD-lipid, was measured by flow cytometry (excitation = 470 nm; emission = 530 nm). Parallel controls included vector-only, ATP8B2 catalytic mutant (D454N), and ATP-depleted cells (treated with 10 mM sodium orthovanadate and 10 mM EDTA). Data were expressed as percent dithionite-resistant fluorescence relative to total signal before quenching and normalized to wild-type ATP8B2 activity. All experiments were performed in triplicate, and results are presented as mean ± SD. Primary TM cells were used for ATP8B2 assay in an identical manner.

Protein–lipid photocrosslinking was performed using a photoactivatable lipid probe (diazirine- or benzophenone-modified phospholipid; Avanti Polar Lipids or synthesized probe) incorporated into membranes either by incubation with live cells or by inclusion (0.5–2 mol%) during liposome formation/reconstitution. Cells or proteoliposomes were equilibrated in ice-cold assay buffer, placed in a clear, uncovered 35–60 mm dish on ice ~5 cm below the Stratalinker light source (Stratagene/Agilent Stratalinker 1800 equipped with 365 nm bulbs), and irradiated at 365 nm (two 60–120 s pulses, total 2–4 min; optimize time empirically to maximize crosslinking while minimizing photodamage). Irradiation on ice minimized thermal drift and non-specific damage; negative controls included samples lacking the photo-probe, non-irradiated samples, and samples containing an inactive protein mutant or non-hydrolyzable nucleotide where appropriate. After UV exposure, reactions were quenched by immediate addition of ice-cold quench buffer (e.g., 50 mM Tris pH 7.5, 150 mM NaCl) and solubilized in SDS sample buffer (or lysed for downstream enrichment), then analyzed by SDS–PAGE followed by in-gel fluorescence (for directly fluorescent probes) or proteomic identification by LC–MS/MS analysis of excised bands. NBD-lipids (NBD-DSPS and NBD-DSPE) was crosslinked with cell extracts in an identical manner as described above and found to work for protein-lipid crosslinking. NBD-Lipid crosslinked proteins separated on 4-15% or IEF 4-9 pH IEF PHAST gels and subjected to LC-MS/MS identification. All steps involving NBD-lipids or photoactivatable reagents were performed protected from ambient light, dithionite/chemical quenching and reducing agents were avoided prior to analysis, and appropriate UV shielding and personal protective equipment were used. Stratalinker bulb type remained unchanged throughout the course of experiments. Key experimental parameters (probe identity and % mol, protein: lipid ratio for reconstitutions, irradiation distance and total exposure, and negative/positive controls) were optimized based on fluorescence estimated using NBD-DSPS and kept unchanged. Three independent observers performed experiments and hence these parameters were recorded for each experiment to ensure reproducibility.

*Statistics.* Data are presented as mean ± SD or mean ± SEM as indicated. Statistical significance and P values were calculated using Student’s 2-tailed t-test or ANOVA, with P < 0.05 and P < 0.01 considered statistically significant, P < 0.001 and P < 0.0001 were considered highly significant. Study designs incorporated randomization. All reagents including any cell line used were subjected to in-house authentication.

*Study approval.* All mice were used in accordance with an approved IACUC protocol of the University of Miami. Human samples/cadaveric tissue procured without identifiers are exempted under NIH category 4. AH samples were procured under University of Miami IRB approved/exempted protocols.

**Data availability**

Lipidomics raw data files, intermediate analysis files and protocols used were submitted to Metabolomics Workbench (<http://www.metabolomicsworkbench.org/>): ST000579-ST000582; ST000612-ST000613. More detailed data and/or protocols are available upon request to corresponding author.

**A reagent checklist/summary**

A reagent list is provided below. We submit this as a manuscript-specific disclosure to document experimental rigor, reproducibility, and transparency for the present study, in alignment with FASEB Journal expectations.

We have used the Power calculation utilizing preliminary data and consulting the experts at Bascom Palmer Biostatistics core. Samples were selected to ensure balanced representation of biological sex. Primary open-angle glaucoma (POAG) patient samples and cadaveric tissues were included only after detailed clinical history review and confirmation that predefined characterization criteria were met. Only samples of high quality and with comprehensive clinical characterization were used for analysis. Samples were processed for profiling experiments largely on a rolling basis as they became available; however, sex distribution and exposure to different glaucoma medication classes were taken into account for patient-derived samples and tissues.

All experiments were conducted under masked conditions. An independent graduate student or senior laboratory staff member, who was not involved in the study and is not a co-author, maintained the coding key in a password-protected system. This individual was solely responsible for managing the code. All collaborators received only coded lipid samples or other reagents, and the identity of the samples remained concealed until completion of data collection and primary analyses.

All raw data of profiling studies have been deposited in Metabolomics workbench.

**Materials and reagents**

1. **Materials availability**

All materials and reagents used in this study, with the exception of patient- or human subject–derived samples, are available without restriction. Lipids employed in the experiments were sourced from commercial suppliers, including Avanti Polar Lipids and Echelon Biosciences, as well as additional vendors (Matreya, Nu-Check and Caymen Biosciences)

1. **Antibodies**

All proteins detected by the antibodies, corresponding to the expected molecular weight bands, were further validated using protein sequencing via nano LC-Q-Exactive tandem mass spectrometry. Because we have access to this instrument in-house, it was routinely used for quality control purposes to confirm the identity of the proteins recognized by the antibodies.

*Western Blots, dot-blots*

| **Antibody** | **Catalog Number** |
| --- | --- |
| Anti-PEMT (phosphatidylethanolamine N- methyltransferase) | Abnova, PAB2194, pAb, LOT SH070924J  catalog number: ABIN543156 |
| Anti- CCTCPT (phosphocholine cytidylyltransferase;) | ab77305 |
| Anti PSD (phosphoserine decarboxylase) | ab93603 |
| Anti EK (ethanolamine kinase) | ab38284 |
| Anti EK (ethanolamine kinase) | ab38284 |
| Anti-PSS1 (phosphatidylserine synthase 1) | Abcam, ab82642, pAb, LOT GR34471-3  catalog number: ab82642 |
| anti-PSS2 (phosphatidylserine synthase 2) | Acris Antibodies, AP53496PU-N, LOT SA110601BJ  catalog number: ABIN954366 |
| Anti-EPT (ethanolamine-phosphotransferase) | catalog number: ABIN1084255 (all from Antibodies Online) |
| anti-GAPDH | Abcam, ab22556, Ab-2302, Millipore, NB600-502 (monoclonal), Novus |
| Lipids: NBD-PS 18:1/18:1 | Avanti, #810225 |
| Anti-ATP8B2 | ab187992 |
| Anti-TMEM30A (Transmembrane Protein 30A) | ab185944 |
| Anti-TMEM30B (Transmembrane Protein 30B) | ab236133 |

Secondary Abs: where applicable we have used high quality fluorephore - HR or other coupled secondary antibodies from Sigma-Aldrich Corporation or from Abcam. These were reviewed and validated antibodies.

*Radioactive isotopes (radioisotopes) used as tracers in biochemical experiments (Revvity Inc., Walthamm MA formerly Perkin Elmer Life Sciences)*

1. (14C) choline

2. (3H) choline

3. 32P

*ELISA for enzymes (antibodies as noted above)*

1. EK (ethanolamine kinase)

2. ECTCTP (phosphoethanolamine cytidylyltransferase)

3. EPT (ethanolamine-phosphotransferase)

4. PSD (phosphoserine decarboxylase)

5. PSS1 (phosphatidylserine synthase 1)

6. PSS2 (phosphatidylserine synthase 2)

7. PEMT (phosphatidylethanolamine N- methyltransferase)

8. CPT (Cholinephosphottransferase)

9. CCTCPT (phosphocholine cytidylyltransferase;)

10. CK (Choline Kinase)

11. ATP8B2

12. TMEM30A

13. TMEM30B

14. PCSK5

*Fluorescent lipid standards*

Avanti usually makes fluorescent lipids by adding a fluorescent tag to the lipid headgroup (like PS or PE), rather than making a fluorescent version of every possible fatty-acid combination. So instead of having a separate product for each exact lipid species (for example, PS with two 18:2 chains), they sell general versions such as fluorescent PS or fluorescent PE. Due to lack of specific products, we had utilized Avanti Polar lipid for custom fluorescent NBD tagged lipid synthesis when needed for the experiment.

| Fluorescent lipid standards | Catalog |
| --- | --- |
| 18:2 PS Closest available: TopFluor™ PS (headgroup labeled) — general TopFluor PS product | 810283 |
| 18:2 PE Closest available: TopFluor™ PE (headgroup labeled) | 810282 |
| 18:0 PS Closest available: TopFluor™ PS (headgroup labeled) — general TopFluor PS product | 810283 |
| 18:1 NBD PS  18:1 PE -TopFluor™ AF488 810386  18:1 PE-TopFluor™ AF594 810387  18:1-06:0 NBD PE 810155  18:1-12:0 NBD PE 810156 | 810198 |
| 18:0 PE TopFluor™ AF488 | 810383 |
| 16:0-06:0 NBD PS  16:0-12:0 NBD PS | 810192  810193 |
| 16:0-06:0 NBD PE  16:0-12:0 NBD PE | 810153  810154 |

We have also obtained isobaric lipid analogs from Matreya, Nu-Check and Caymen Biosciences. A few of these involved custom synthesis.

**PSD enzymatic activity assay**

We utilized following reagents for positive/negative controls:

lentiviral-mediated PSD overexpression (Genecopoeia Inc.)

CRISPR-Cas9 depletion of ATP8B2 in primary TM cells: Genecopoeia Inc.

1. **Eukaryotic cell lines**

The following eukaryotic cell lines were used in this study:

1. HEK293T (sourced from ATCC)
2. HTM-5 (provided by Alcon Laboratories, Fort Worth, TX, authenticated using recommended methods: see below)
3. Primary trabecular meshwork (TM) cells isolated from donor tissues using published protocols.

Cell line authentication was performed as follows:

1. TM-derived cell lines were validated by assessing myocilin overexpression in response to dexamethasone treatment
2. Primary TM cells were authenticated using the same approach.
3. HEK293T cells were authenticated by ATCC.

All cell lines were routinely tested for mycoplasma contamination using standard laboratory procedures, primarily with the Thermo Fisher C7028 Mycoplasma Detection Kit, and on select occasions with the MycoFluor™ Mycoplasma Detection Kit (catalog M7006) provided by Dr. Darlene Miller (Bascom Palmer Eye Institute).

No cell lines listed in the ICLAC database of commonly misidentified lines were used in this study.

**Animals and human research participants**

**A. Research animals**

| **Animals** | **Details** |
| --- | --- |
| DBA2J mice | 6-9 months old, equal sex distribution from Jackson Labs, Bar Harbor, Maine. |
| DBA/2J-Gpnmb + /SjJ mice | - 1. months old, equal sex distribution from Jackson Labs, Bar Harbor, Maine. |

Mice were genotyped by Taconic Biosciences Inc. as service using appropriate primers for authentication purposes.

**B. Human research participants**

Description of donors with full details has been provided as **Supplementary Table 1.**

**Data analysis software**

| Microsoft Office Excel | Custom MATLAB software |
| --- | --- |
| GraphPad Prism | MFP-3D AFM system software based in IGOR Pro |
| Adobe Illustrator | Dynamics 5.26.02 |
| Lipid Search 4.1 | pClamp 10 |
| Xcalibur 2.3 | Clampfit 10 |
| Chipsoft 2.3 | Qimaging Image Proplus |
| MZmine 2.9 | ImageJ |
| OcuMetrics | Custom MATLAB-based software for OCT analysis |
| PowerLab | Proteome Discoverer 3.2 |

**References**

Aihara, M., Lindsey, J.D., and Weinreb, R.N. (2002). Reduction of intraocular pressure in mouse eyes treated with latanoprost. Invest Ophthalmol Vis Sci *43*, 146-150.

Aihara, M., Lindsey, J.D., and Weinreb, R.N. (2003). Episcleral venous pressure of mouse eye and effect of body position. Curr Eye Res *27*, 355-362.

Aljohani, A.J., Edwards, G., Guerra, Y., Dubovy, S., Miller, D., Lee, R.K., and Bhattacharya, S.K. (2014). Human trabecular meshwork sphingolipid and ceramide profiles and potential latent fungal commensalism. Invest Ophthalmol Vis Sci *55*, 3413-3422.

Aljohani, A.J., Munguba, G.C., Guerra, Y., Lee, R.K., and Bhattacharya, S.K. (2013). Sphingolipids and ceramides in human aqueous humor. Mol Vis *19*, 1966-1984.

Amelinckx, A., Castello, M., Arrieta-Quintero, E., Lee, T., Salas, N., Hernandez, E., Lee, R.K., Bhattacharya, S.K., and Parel, J.M. (2009). Laser trabeculoplasty induces changes in the trabecular meshwork glycoproteome: a pilot study. J Proteome Res *8*, 3727-3736.

Aribindi, K., Guerra, Y., Lee, R.K., and Bhattacharya, S.K. (2013). Comparative phospholipid profiles of control and glaucomatous human trabecular meshwork. Invest Ophthalmol Vis Sci *54*, 3037-3044.

Barbosa, A.J., Castro, L.P., Margarida, A., and Nogueira, M.F. (1984). A simple and economical modification of the Masson-Fontana method for staining melanin granules and enterochromaffin cells. Stain Technol *59*, 193-196.

Bhattacharya, S.K. (2013). Recent advances in shotgun lipidomics and their implication for vision research and ophthalmology. Curr Eye Res *38*, 417-427.

Bhattacharya, S.K., Gabelt, B.T., Ruiz, J., Picciani, R., and Kaufman, P.L. (2009). Cochlin expression in anterior segment organ culture models after TGFbeta2 treatment. Invest Ophthalmol Vis Sci *50*, 551-559.

Bhattacharya, S.K., Rockwood, E.J., Smith, S.D., Bonilha, V.L., Crabb, J.S., Kuchtey, R.W., Robertson, N.G., Peachey, N.S., Morton, C.C., and Crabb, J.W. (2005). Proteomics reveals cochlin deposits associated with glaucomatous trabecular meshwork. J Biol Chem *280*, 6080-6084.

Bligh, E.G., and Dyer, W.J. (1959). A rapid method of total lipid extraction and purification. Can J Biochem Physiol *37*, 911-917.

Boussommier-Calleja, A., and Overby, D.R. (2013). The influence of genetic background on conventional outflow facility in mice. Invest Ophthalmol Vis Sci *54*, 8251-8258.

Bradford, M.M. (1976). A rapid and sensitive method for the quantitation of microgram quantities of protein utilizing the principle of protein-dye binding. Anal Biochem *72*, 248-254.

Brewer, G.J., Torricelli, J.R., Evege, E.K., and Price, P.J. (1993). Optimized survival of hippocampal neurons in B27-supplemented Neurobasal, a new serum-free medium combination. J Neurosci Res *35*, 567-576.

Camras, L.J., Sufficool, K.E., Camras, C.B., Fan, S., Liu, H., and Toris, C.B. (2010). Duration of anesthesia affects intraocular pressure, but not outflow facility in mice. Curr Eye Res *35*, 819-827.

Carreon, T., van der Merwe, E., Fellman, R.L., Johnstone, M., and Bhattacharya, S.K. (2016a). Aqueous outflow - A continuum from trabecular meshwork to episcleral veins. Prog Retin Eye Res, In press.

Carreon, T.A., Edwards, G., Wang, H., and Bhattacharya, S.K. (2016b). Segmental outflow of aqueous humor in mouse and human. Exp Eye Res, In press.

Chatterjee, A., Oh, D.J., Kang, M.H., and Rhee, D.J. (2013). Central corneal thickness does not correlate with TonoLab-measured IOP in several mouse strains with single transgenic mutations of matricellular proteins. Exp Eye Res *115*, 106-112.

Chou, T.H., Bohorquez, J., Toft-Nielsen, J., Ozdamar, O., and Porciatti, V. (2014). Robust mouse pattern electroretinograms derived simultaneously from each eye using a common snout electrode. Invest Ophthalmol Vis Sci *55*, 2469-2475.

Edwards, G., Aribindi, K., Guerra, Y., and Bhattacharya, S.K. (2014a). Sphingolipids and ceramides of mouse aqueous humor: Comparative profiles from normotensive and hypertensive DBA/2J mice. Biochimie *105*, 99-109.

Edwards, G., Aribindi, K., Guerra, Y., Lee, R.K., and Bhattacharya, S.K. (2014b). Phospholipid profiles of control and glaucomatous human aqueous humor. Biochimie *101*, 232-247.

Enriquez-Algeciras, M., and Bhattacharya, S.K. (2013). Lipidomic mass spectrometry and its application in neuroscience. World J Biol Chem *4*, 102-110.

Goel, M., Sienkiewicz, A.E., Picciani, R., Lee, R.K., and Bhattacharya, S.K. (2011). Cochlin induced TREK-1 co-expression and annexin A2 secretion: role in trabecular meshwork cell elongation and motility. PloS one *6*, e23070.

Goel, M., Sienkiewicz, A.E., Picciani, R., Wang, J., Lee, R.K., and Bhattacharya, S.K. (2012). Cochlin, intraocular pressure regulation and mechanosensing. PloS one *7*, e34309.

Govindarajan, B., Laird, J., Salomon, R.G., and Bhattacharya, S.K. (2008). Isolevuglandin-modified proteins, including elevated levels of inactive calpain-1, accumulate in glaucomatous trabecular meshwork. Biochemistry *47*, 817-825.

Guerra, Y., Aljohani, A.J., Edwards, G., and Bhattacharya, S.K. (2014). A comparison of trabecular meshwork sphingolipids and ceramides of ocular normotensive and hypertensive states of DBA/2J mice. J Ocul Pharmacol Ther *30*, 283-290.

Guerra, Y., Aribindi, K., Edwards, G., Olivos, H.J., Bahrainwala, T.M., Mandal, N.A., and Bhattacharya, S.K. (2015). An overview of Sphingolipids and ceramides of aqueous humor and trabecular meshwork. In Sphingolipids: Biology, Synthesis and Functions, A. Catala, ed. (Hauppauge, New York: Nova Science Publishers, Inc.).

Helwa, I., Cai, J., Drewry, M.D., Zimmerman, A., Dinkins, M.B., Khaled, M.L., Seremwe, M., Dismuke, W.M., Bieberich, E., Stamer, W.D.*, et al.* (2017). A Comparative Study of Serum Exosome Isolation Using Differential Ultracentrifugation and Three Commercial Reagents. PloS one *12*, e0170628.

John, S.W., Hagaman, J.R., MacTaggart, T.E., Peng, L., and Smithes, O. (1997). Intraocular pressure in inbred mouse strains. Invest Ophthalmol Vis Sci *38*, 249-253.

Khatun, Z., Bhat, A., Sharma, S., and Sharma, A. (2016). Elucidating diversity of exosomes: biophysical and molecular characterization methods. Nanomedicine (Lond) *11*, 2359-2377.

Millar, J.C., Clark, A.F., and Pang, I.H. (2011). Assessment of aqueous humor dynamics in the mouse by a novel method of constant-flow infusion. Invest Ophthalmol Vis Sci *52*, 685-694.

Millar, J.C., Phan, T.N., Pang, I.H., and Clark, A.F. (2015). Strain and Age Effects on Aqueous Humor Dynamics in the Mouse. Invest Ophthalmol Vis Sci *56*, 5764-5776.

Pang, I.H., Shade, D.L., Clark, A.F., Steely, H.T., and DeSantis, L. (1994). Preliminary characterization of a transformed cell strain derived from human trabecular meshwork. Curr Eye Res *13*, 51-63.

Picciani, R., Desai, K., Guduric-Fuchs, J., Cogliati, T., Morton, C.C., and Bhattacharya, S.K. (2007). Cochlin in the eye: functional implications. Prog Retin Eye Res *26*, 453-469.

Porciatti, V. (2007). The mouse pattern electroretinogram. Doc Ophthalmol *115*, 145-153.

Russell, P., and Johnson, M. (2012). Elastic modulus determination of normal and glaucomatous human trabecular meshwork. Invest Ophthalmol Vis Sci *53*, 117.

Savinova, O.V., Sugiyama, F., Martin, J.E., Tomarev, S.I., Paigen, B.J., Smith, R.S., and John, S.W. (2001). Intraocular pressure in genetically distinct mice: an update and strain survey. BMC Genet *2*, 12.

Shah, N., Ishii, M., Brandon, C., Ablonczy, Z., Cai, J., Liu, Y., Chou, C.J., and Rohrer, B. (2018). Extracellular vesicle-mediated long-range communication in stressed retinal pigment epithelial cell monolayers. Biochim Biophys Acta *1864*, 2610-2622.

Shaner, R.L., Allegood, J.C., Park, H., Wang, E., Kelly, S., Haynes, C.A., Sullards, M.C., and Merrill, A.H., Jr. (2009). Quantitative analysis of sphingolipids for lipidomics using triple quadrupole and quadrupole linear ion trap mass spectrometers. J Lipid Res *50*, 1692-1707.

Smith, R.S., John, S.W.M., Nishina, P.M., and Sundberg, J.P. (2002). Systematic evaluation of the mouse eye. . In Systematic Approach to Evaluation of Mouse Mutations., J.P. Sundberg, and D. Boggess, eds. (Boca Raton, Florida: CRC Press).

Stamer, W.D., Seftor, R.E., Williams, S.K., Samaha, H.A., and Snyder, R.W. (1995). Isolation and culture of human trabecular meshwork cells by extracellular matrix digestion. Curr Eye Res *14*, 611-617.

Thery, C., Amigorena, S., Raposo, G., and Clayton, A. (2006). Isolation and characterization of exosomes from cell culture supernatants and biological fluids. Curr Protoc Cell Biol *Chapter 3*, Unit 3 22.

Toris, C.B., Fan, S., Johnson, T.V., Camras, L.J., Hays, C.L., Liu, H., and Ishimoto, B.M. (2016). Aqueous Flow Measured by Fluorophotometry in the Mouse. Invest Ophthalmol Vis Sci *57*, 3844-3852.

Wang, H., Edwards, G., Garzon, C., Piqueras, C., and Bhattacharya, S.K. (2015). Aqueous humor phospholipids of DBA/2J and DBA/2J-Gpnmb(+)/SjJ mice. Biochimie *113*, 59-68.

Yablonski, M.E., Zimmerman, T.J., Waltman, S.R., and Becker, B. (1978). A fluorophotometric study of the effect of topical timolol on aqueous humor dynamics. Exp Eye Res *27*, 135-142.

Yang, K., Cheng, H., Gross, R.W., and Han, X. (2009). Automated lipid identification and quantification by multidimensional mass spectrometry-based shotgun lipidomics. Anal Chem *81*, 4356-4368.
