# Supplementary material for: Phospholipid Interconversion and Transport Are Altered in Glaucoma
Source: FASEB J. 2026 Jan 20;40(2):e71451. doi: 10.1096/fj.202504068R (PMC12817104; doi:10.1096/fj.202504068R)
Supplement: Supplementary file 2 — Figure S1: Representative confirmation of lipids via fragment ion signatures and differences in phospholipid transport in the POAG TM. Figure S2: Vast difference in fold change in several similar phospholipids in the TM in POAG. Figure S3: PSD level compared to beta actin. Figure S4: Probing the ATP8B2 blots in Figure 2 with anti‐CD133 and anti‐beta actin. Figure S5:. Probing Figure 3 animal model proteins for ATP8B2 and PSD. [file FSB2-40-e71451-s001.pptx]

## Slide 1
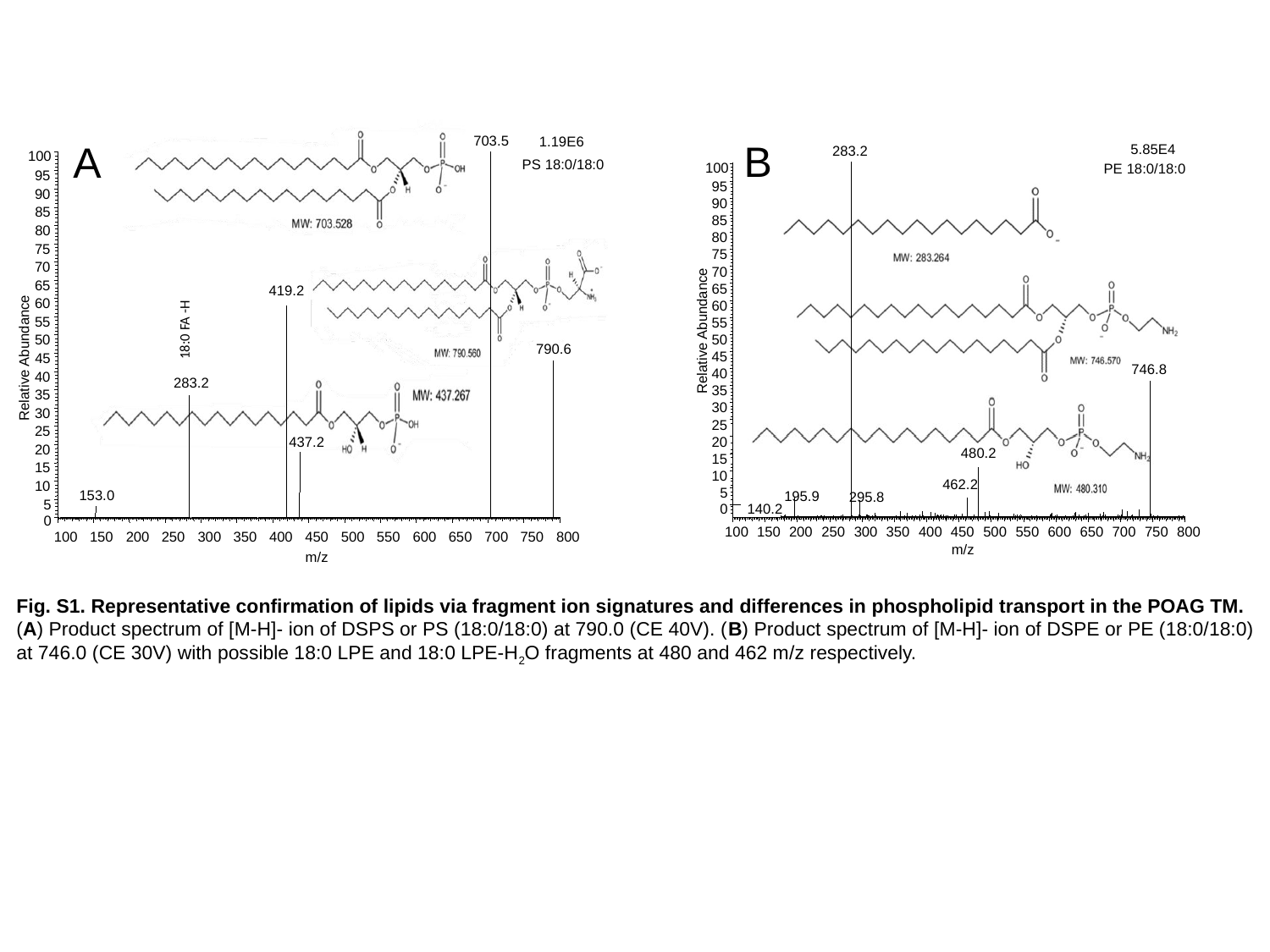

A
703.5
1.19E6
PS 18:0/18:0
100
95
90
85
18:0 FA -H
80
75
70
65
419.2
60
55
Relative Abundance
50
790.6
45
40
283.2
35
30
25
437.2
20
15
10
153.0
5
0
100
150
200
250
300
350
400
450
500
550
600
650
700
750
800
m/z
B
5.85E4
283.2
PE 18:0/18:0
100
95
90
85
80
75
70
65
60
55
Relative Abundance
50
45
40
35
30
25
20
15
10
5
0
100
150
200
250
300
350
400
450
500
550
600
650
700
750
800
m/z
746.8
480.2
462.2
195.9
295.8
140.2
Fig. S1. Representative confirmation of lipids via fragment ion signatures and differences in phospholipid transport in the POAG TM. (A) Product spectrum of [M-H]- ion of DSPS or PS (18:0/18:0) at 790.0 (CE 40V). (B) Product spectrum of [M-H]- ion of DSPE or PE (18:0/18:0) at 746.0 (CE 30V) with possible 18:0 LPE and 18:0 LPE-H2O fragments at 480 and 462 m/z respectively.

## Slide 2
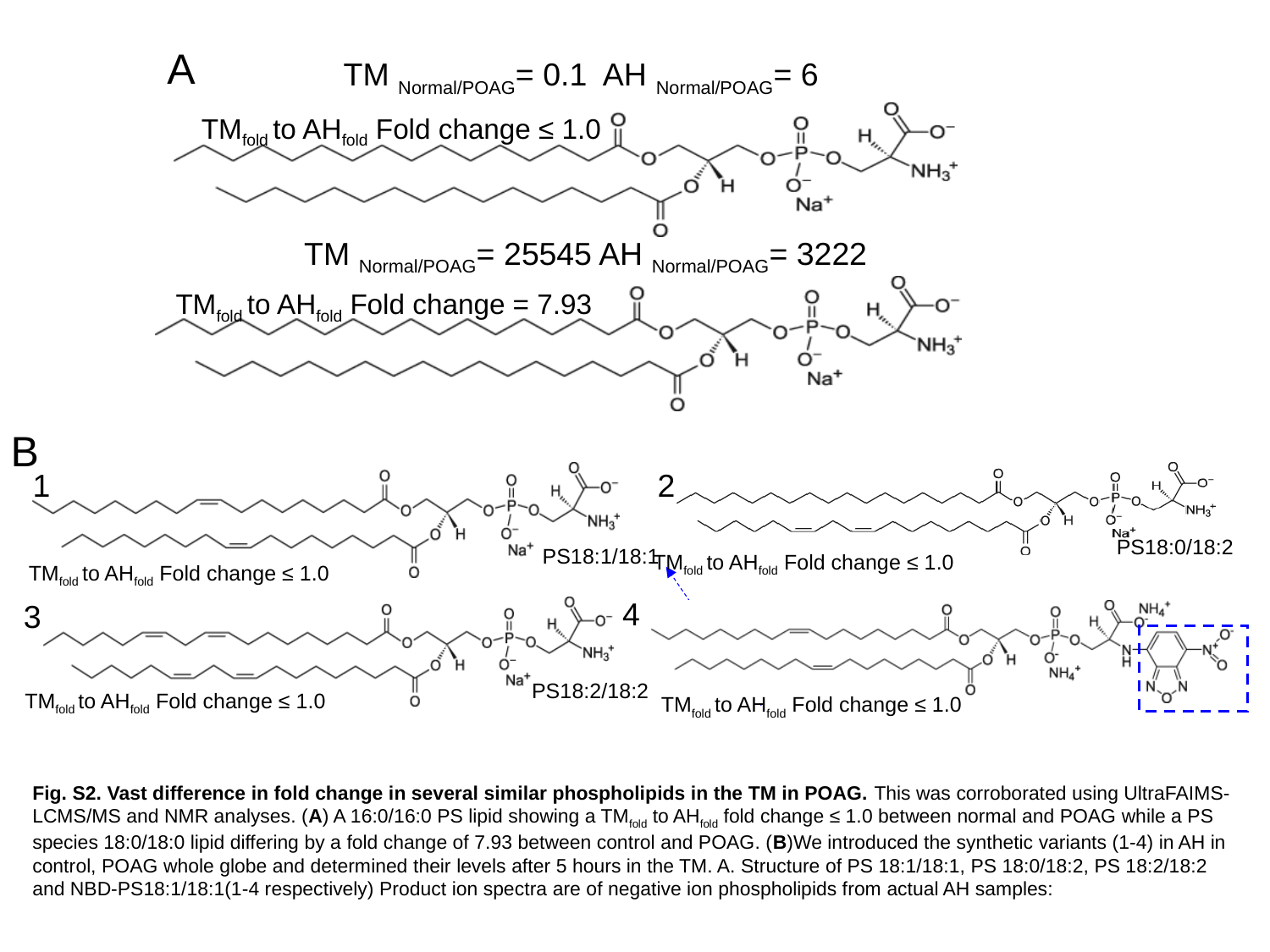

A
TM Normal/POAG= 0.1 AH Normal/POAG= 6
TMfold to AHfold Fold change ≤ 1.0
TM Normal/POAG= 25545 AH Normal/POAG= 3222
TMfold to AHfold Fold change = 7.93
B
1
2
PS18:0/18:2
PS18:1/18:1
TMfold to AHfold Fold change ≤ 1.0
TMfold to AHfold Fold change ≤ 1.0
4
3
PS18:2/18:2
TMfold to AHfold Fold change ≤ 1.0
TMfold to AHfold Fold change ≤ 1.0
Fig. S2. Vast difference in fold change in several similar phospholipids in the TM in POAG. This was corroborated using UltraFAIMS-LCMS/MS and NMR analyses. (A) A 16:0/16:0 PS lipid showing a TMfold to AHfold fold change ≤ 1.0 between normal and POAG while a PS species 18:0/18:0 lipid differing by a fold change of 7.93 between control and POAG. (B)We introduced the synthetic variants (1-4) in AH in control, POAG whole globe and determined their levels after 5 hours in the TM. A. Structure of PS 18:1/18:1, PS 18:0/18:2, PS 18:2/18:2 and NBD-PS18:1/18:1(1-4 respectively) Product ion spectra are of negative ion phospholipids from actual AH samples:

## Slide 3
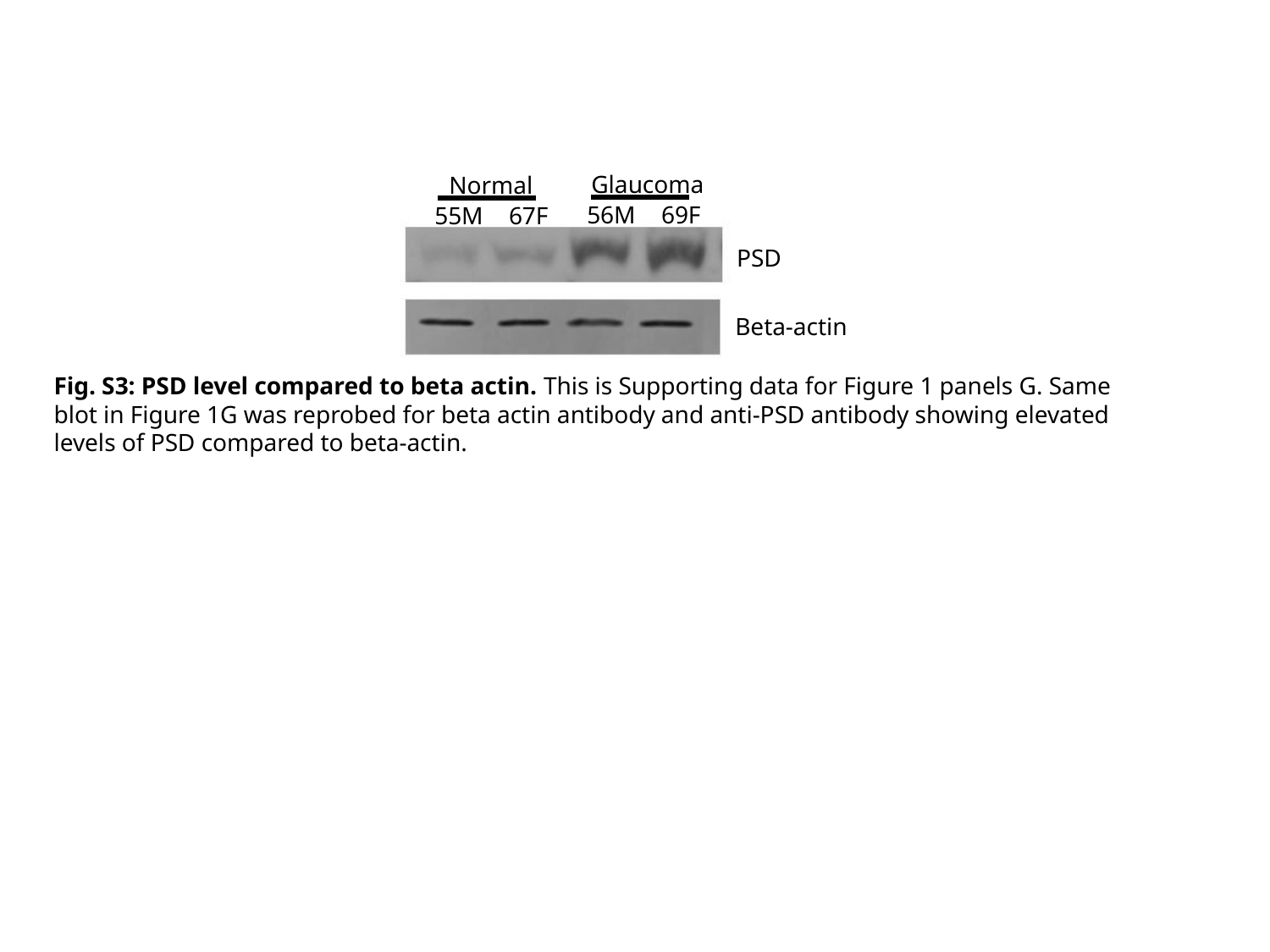

Glaucoma
Normal
56M
69F
55M
67F
PSD
Beta-actin
Fig. S3: PSD level compared to beta actin. This is Supporting data for Figure 1 panels G. Same blot in Figure 1G was reprobed for beta actin antibody and anti-PSD antibody showing elevated levels of PSD compared to beta-actin.

## Slide 4
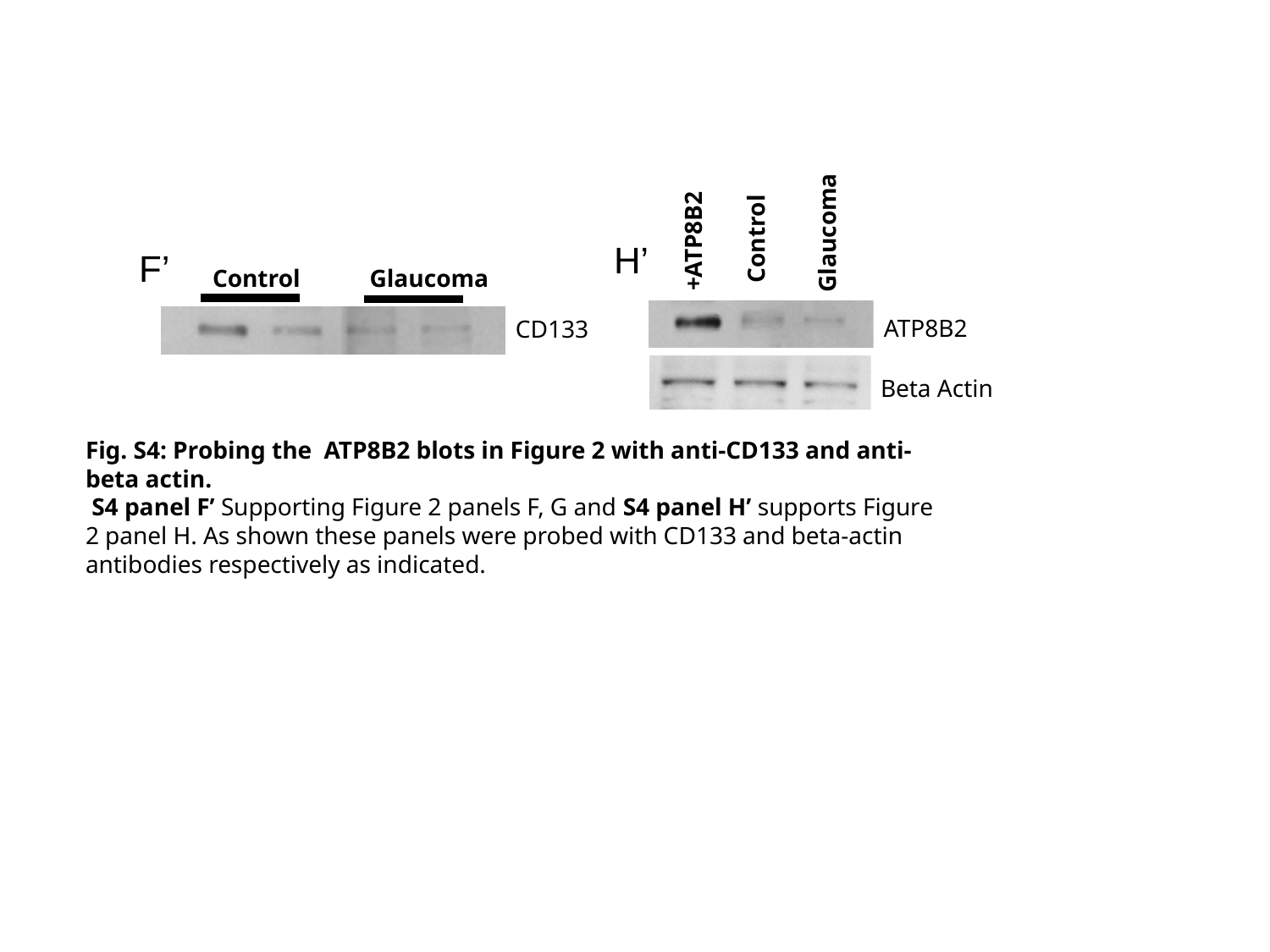

Glaucoma
Control
+ATP8B2
H’
F’
Control
Glaucoma
ATP8B2
CD133
Beta Actin
Fig. S4: Probing the ATP8B2 blots in Figure 2 with anti-CD133 and anti-beta actin.
 S4 panel F’ Supporting Figure 2 panels F, G and S4 panel H’ supports Figure 2 panel H. As shown these panels were probed with CD133 and beta-actin antibodies respectively as indicated.

## Slide 5
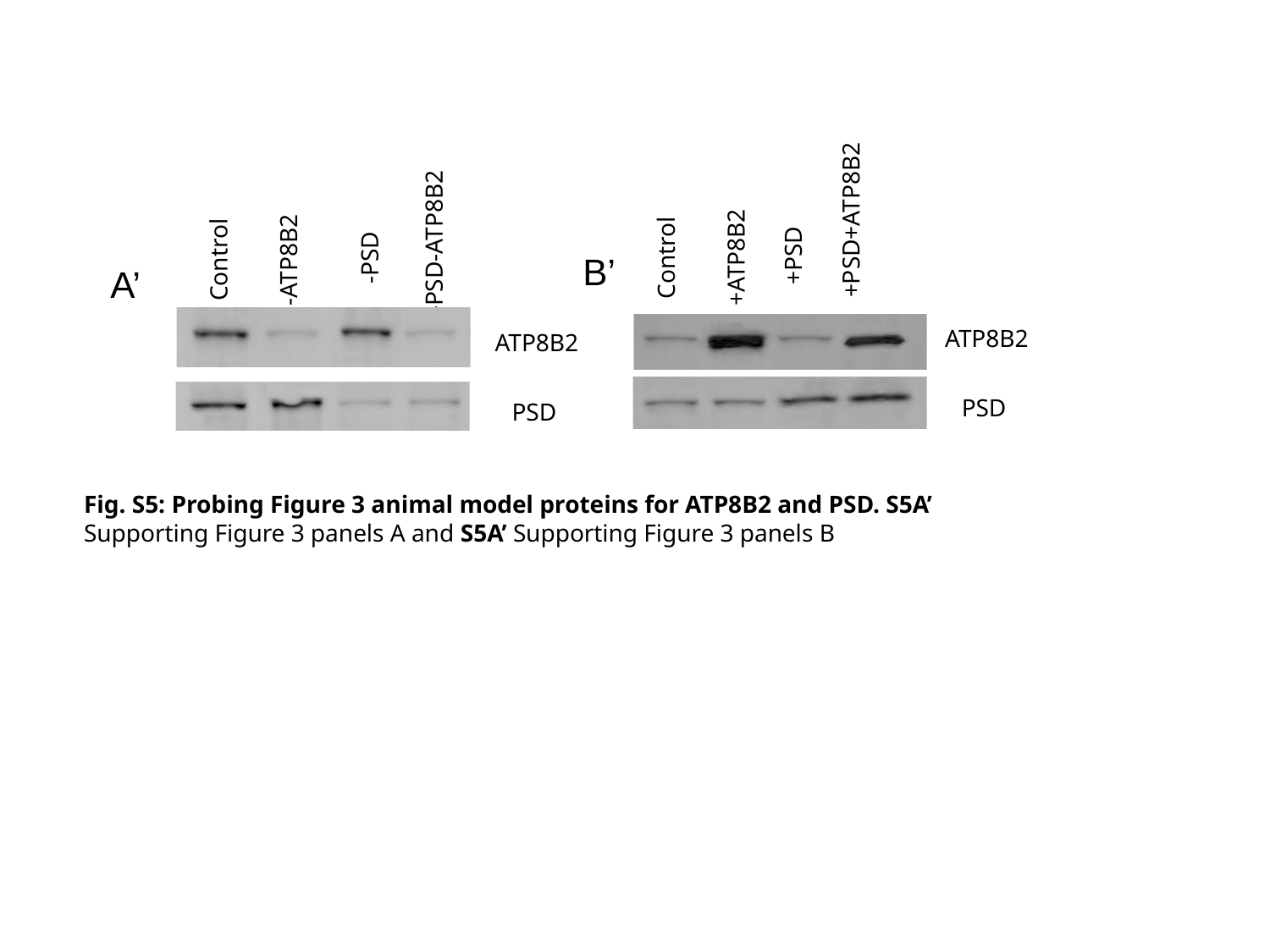

+PSD+ATP8B2
-PSD-ATP8B2
+PSD
-PSD
+ATP8B2
Control
-ATP8B2
Control
B’
A’
ATP8B2
ATP8B2
PSD
PSD
Fig. S5: Probing Figure 3 animal model proteins for ATP8B2 and PSD. S5A’ Supporting Figure 3 panels A and S5A’ Supporting Figure 3 panels B
